# Analytical Profile and Antioxidant and Anti-Inflammatory Activities of the Enriched Polyphenol Fractions Isolated from Bergamot Fruit and Leave

**DOI:** 10.3390/antiox10020141

**Published:** 2021-01-20

**Authors:** Giovanna Baron, Alessandra Altomare, Marco Mol, Jessica Leite Garcia, Camila Correa, Angela Raucci, Luigi Mancinelli, Sarah Mazzotta, Laura Fumagalli, Giuseppe Trunfio, Luigi Tucci, Elena Lombardo, Domenico Malara, Elzbieta Janda, Vincenzo Mollace, Marina Carini, Ezio Bombardelli, Giancarlo Aldini

**Affiliations:** 1 Department of Pharmaceutical Sciences, University of Milan, 20133 Milan, Italy; giovanna.baron@unimi.it (G.B.); alessandra.altomare@unimi.it (A.A.); marcomol89@gmail.com (M.M.); jessleitegarcia@gmail.com (J.L.G.); sarah.mazzotta@unimi.it (S.M.); laura.fumagalli@unimi.it (L.F.); marina.carini@unimi.it (M.C.); 2 Medical School, Sao Paulo State University (Unesp), Botucatu 18618-687, Brazil; correa.camila9@gmail.com; 3 Experimental Cardio-oncology and Cardiovascular Aging Unit, Centro Cardiologico Monzino-IRCCS, Via Carlo Parea, 4, 20138 Milan, Italy; angela.raucci@cardiologicomonzino.it (A.R.); jkducati695@gmail.com (L.M.); 4 H&AD Srl, 89032 Bianco, Italy; g.trunfio@head-sa.com (G.T.); l.tucci@head-sa.com (L.T.); e.lombardo@head-sa.com (E.L.); d.malara@head-sa.com (D.M.); 5 Department of Health Sciences, University “Magna Graecia” of Catanzaro, 88100 Catanzaro, Italy; janda@unicz.it (E.J.); mollacev@gmail.com (V.M.); 6 Plantexresearch Srl, 20122 Milan, Italy; ezio.bombardelli@plantexresearch.it

**Keywords:** *Citrus bergamia*, polyphenols, high-resolution mass spectrometry, antioxidant, inflammation, metabolic syndrome, cholesterol, HMG

## Abstract

The aim of the study is to compare the qualitative and semi-quantitative profile of the polyphenol fraction purified from the leaf (BLPF) and fruit (BFPF) of bergamot (*Citrus bergamia*), and to evaluate their antioxidant and anti-inflammatory activity. The analytical qualitative profile was carried out by LC-ESI/MS using three different approaches: targeted (searching analytes already reported in bergamot extract), semi-targeted (a selective search of 3-hydroxy-3-methylglutarate [HMG] derivatives involved in the cholesterol reducing activity of BPF) and untargeted. A total number of 108 compounds were identified by using the three approaches, 100 of which are present in both the extracts thus demonstrating a good qualitative overlapping of polyphenols between the two extracts. The antioxidant activity was higher for BLPF in respect to BFPF but when normalized in respect to the polyphenol content they were almost overlapping. Both the extracts were found to dose dependently inhibit cell inflammation stimulated with IL-1α. In conclusion, the comparison of the qualitative and quantitative profile of polyphenols as well as of the antioxidant and anti-inflammatory activity of bergamot leaf and fruit well indicates that leaf is a valid source of bergamot polyphenol extraction and an even richer source of polyphenol in respect to the fruit.

## 1. Introduction

The bergamot (*Citrus bergamia*), is an ancient fruit bearing tree used for the production of its essential oil. Although native to South-East Asia, the plant is also grown in Italy where due to the fact that it is very sensitive to the pedoclimatic conditions of the soil, bergamot cultivation is currently limited to the coastal area of Calabria (southern Italy), from Reggio Calabria to Locri, where the climate and environmental conditions are favorable to its cultivation. Italy accounts for over 95% of world production of bergamot essential oil which is obtained from the peel and is widely used in the cosmetics industry [1,2,3].

Besides the commercial and scientific interest in the essential oil, in recent years the scientific community has focused growing attention on the juice of bergamot which has interesting nutraceutical potential as reviewed in some recent papers [4,5,6]. In particular, several pharmacological and intervention studies have reported that bergamot juice or rather its enriched polyphenolic fraction (BPF), obtained from the peeled fruit and mainly composed of flavanones (such as naringenin, hesperetin, eriodictyol glycosides), flavones, (apigenin, luteolin, chrysoeriol, diosmetin glycosides) and their 3-hydroxy-3-methyl-glutaryl (HMG) derivatives, has hypolipemic, hypoglycemic, and anti-inflammatory activities and, more generally, is effective in the treatment of metabolic syndrome symptoms [7,8,9,10]. Such in vivo effects have been linked to the antioxidant and anti-inflammatory activities of its constituents and to the ability of HMG derivatives, such as melitidin and brutieridin, to bind the catalytic site of HMG-CoA reductase and inhibit cholesterol synthesis by replacing its endogenous substrate HMG-CoA [11,12].

Based on the growing scientific evidence of the positive effects of BPF on human health, we believe that in the next few years there will be an increased demand for BPF as a supplementary ingredient. However, considering the limited availability of the fruit due to the restricted area of bergamot cultivation, the increased demand for the bergamot polyphenols should be addressed by considering an alternative source which must have a qualitative and quantitative composition and biological activity overlapping those of the fruit.

Analytical studies show that for some plants, such as some of those which produce berries, the phenolic composition of the leaf is similar to that of the precious fruit or even richer and higher, indicating that they may be utilized as an alternative source of bioactive natural products for the development of food supplements, nutraceuticals, or functional foods [13].

The aim of the paper is to fully analyze and compare the qualitative and semi-quantitative profile of enriched polyphenol fraction from bergamot leaf (BLPF) and fruit (BFPF) and prepared by using the same process so that their qualitative and quantitative profile can be compared. The two extracts would then be evaluated in terms of antioxidant and anti-inflammatory activity. The results will permit us to understand whether bergamot leaves can be considered as an alternative plant source to the fruit for the extraction of polyphenols to be used as food supplements and nutraceuticals.

## 2. Materials and Methods

### 2.1. Reagents

6-hydroxy-2,5,7,8-tetramethyl-3,4-dihydrochromene-2-carboxylic acid (Trolox, cat. number 238813), DMSO (cat. number 472301), formic acid (cat. number 00940), ammonium acetate (cat. number 238074), Sephadex™ LH-20 (cat. number GE17-0090), bradykinin acetate (cat. number B3259), tannic acid (cat. number 403040), Folin-Ciocalteu reagent (cat. number 47641), sodium carbonate (cat. number 223530), gallic acid (cat. number G7384), 2′,7′-dichlorofluorescein (cat. number 410217), sodium phosphate dibasic (cat. number S9763), sodium phosphate monobasic monohydrate (cat. number S9638), 2,2′-azobis(2-methylpropionamidine) dihydrochloride (ABAP, cat. number 440914), 2,2′-azino-bis (3-ethylbenzothiazoline-6-sulfonic acid) diammonium salt (ABTS, cat. number A1888), potassium persulfate (cat. number 216224), sodium acetate (cat. number 241245), acetic acid (cat. number 695092), 2,2-diphenyl-1-picrylhydrazyl (DPPH, cat. number D9132), 3-(4,5-Dimethyl-2-thiazolyl)-2,5-diphenyl-2H-tetrazolium bromide (MTT, cat. number M2128), IL-1 α (cat. number SRP3310) and LC–MS grade solvents were purchased from Merck KGaA, Darmstadt, Germany. The peptide LVNEVTEF was custom synthesized by Sigma-Aldrich (Milan, Italy). LC-grade H_2_O (18 MΩ cm) was prepared with a Milli-Q H_2_O purification system (Millipore, Bedford, MA, USA).

### 2.2. Plant Material

#### 2.2.1. BFPF Preparation

The albedo of bergamot fruit is minced with water in order to extract polyphenols, and to this the mixture is added a pectolytic enzyme to decrease the viscosity by degrading the pectin. The fluid is then clarified by means of an ultrafiltration process with semipermeable membranes having a selectivity equal to 12,000 Da. The solution is then passed through a polystirenic absorbing resin bed with pores of 100–150 Angstroms diameter. The entrapped polyphenols are then eluted by modifying their external conformation making the pH basic. Since the polyphenols in this form are unstable, they are passed through a cationic resin bed to re-establish the natural acid pH of 2.0–3.0. The obtained water solution is evaporated under vacuum at temperatures up to 60 °C for less than 1 min until a concentrated water solution with a total polyphenol concentration of 40% is obtained. This is then dried in a spray dryer system thus obtaining a powder with less than 4.0% of humidity.

#### 2.2.2. BLPF Preparation

The bergamot leaves are harvested and subsequently minced and a water/ethanol (30/70, %*v*/*v*) solution is added to the extract the polyphenols. The ethanol is then distilled, and the clarified water solution is passed through a polystirenic absorbing resin bed having with pores of 100–150 Angstroms diameter. The entrapped polyphenols are then eluted with pure ethanol. The obtained ethanolic solution is distilled at temperatures up to 40 °C obtaining a concentrated containing residual water with polyphenols, which is then dried in a spray dryer system obtaining a powder with less than 4.0% of humidity.

### 2.3. LC-HR-MS Conditions

The stock solutions (2.5 mg/mL) of the two bergamot extracts were prepared by dissolving the powder in methanol, then diluted 1:4 in H_2_O/HCOOH, 100/0.1, %*v*/*v* (mobile phase A) and spiked with the internal standard (6-hydroxy-2,5,7,8-tetramethyl-3,4-dihydrochromene-2-carboxylic acid) at a final concentration of 50 µM. Each sample (20 µL) was analyzed in triplicate by LC-HRMS as described by Baron et al. with few modifications [14]. Briefly, the chromatographic separation was performed using an RP Agilent Zorbax SB-C18 column (150 × 2.1 mm, i.d. 3.5 µm, CPS analitica, Milan, Italy) by an UltiMate 3000 system (Dionex) with a multistep program (80 min) of mobile phase A (H_2_O/HCOOH, 100/0.1, %*v*/*v*) and B (CH_3_CN/HCOOH, 100/0.1, %*v*/*v*). An LTQ Orbitrap XL mass spectrometer equipped with an ESI source was used as analyzer, working in data dependent scan mode: three different collision energies (CID) 10, 20 and 40 eV were used to fragment each ion selected in the full MS scan to obtain the best fragmentation pattern for each type of metabolite. The spectra were acquired in negative ion mode. Xcalibur 4.0 and Chromeleon Xpress 6.80 were used for instrument control and spectra analysis.

### 2.4. Targeted, Semi-Targeted and Untargeted Analysis of Bergamot Extract Components

A database (Table S1 of Supplementary Materials) was built searching in the literature for the known bergamot components [9,15,16,17,18,19,20,21,22,23,24,25]. The targeted analysis was performed by searching for all the components listed in the database on the basis of their exact mass ([M – H]^−^), with a mass tolerance of 5 ppm. The fragmentation pattern was used to confirm the identity. A semi-targeted approach was designed to identify the most intense HMG derivatives according to the peculiar mass losses of 3-hydroxy-3-methylglutarate (HMG): three ion maps were generated on the Qual Browser of Xcalibur by selecting the three neutral losses: 62, 102 and 144 Da, with a mass tolerance of 0.3 Da. Three lists of precursor ions were obtained and cross-checked to select the common ions. The fragmentation of the precursor ions was then manually verified to confirm the presence of the three losses. The hypotheses of identification were performed by using the Qual Browser Elemental Composition tool of Xcalibur with the following settings: charge −1, mass tolerance 5 ppm, elements in use C < 60, H < 100, O < 40, N < 2. The formulae thus obtained were compared to that of the structure hypothesized by the fragmentation. The untargeted analysis was performed by searching for the ions (intensity > 10^4^ counts) not identified using the previous methods. The top 5 molecular formulae calculated as for the semi-targeted approach were searched for in the available databases (HMDB, MoNa, PubChem, etc) to find candidates. The experimental MS/MS spectra were compared to those generated in silico by the Peak Assignment tool of CFM-ID online software to verify the identity (fragment tolerance 0.3 Da). The similarity search tool of MoNa was also used to annotate the unknown compounds as well as the Compound Identification tool of CFM-ID, using as mass tolerance 5 ppm for the precursor ion and 0.3 Da for the fragments. Finally, some losses were used to hypothesize structures not identified with the previous methods and were then confirmed by the Peak Assignment tool of CFM-ID: −162 for O-glucoside, −120 for C-glucoside, −146 for O-rhamnoside, −42 for acetyl moiety.

### 2.5. Semi-Quantitative Data Analysis

A semi-quantitative analysis of each identified metabolite was carried out by reconstituting the corresponding single ion chromatogram (SIC) by setting the molecular ion as filter ion and a tolerance of 5 ppm. The area under the curve of each metabolite was then automatically integrated as was that of the internal standard (Trolox). The ratio between the AUC of each metabolite (AUC_n_) and the AUC of the IS (AUC_IS_) was then calculated and divided by the sum of the ratios of all the compounds and expressed as % as reported by the equation (1)

(1) (AUCnAUCIS∑ AUCnAUCIS)×10

Volcano plot was built by plotting, for each identified analyte, on *x* axis, the log2 of the fold change between (AUC_n_)/(AUC_IS_)BLPF vs. (AUC_n_)/(AUC_IS_)BFPF and on the *y* axis the -Log *p* value of the mean ratios. Those metabolites having a log2 fold change ≥ 1 or ≤ −1 and -Log *p* value ≥ 2.5 were considered to have a relative content significantly different in the two extracts.

### 2.6. Quantitative Analysis of Selected Bergamot Components

The major flavonoids (neoeriocitrin, naringin, neohesperidin, melitidin, and brutieridin) present in bergamot extracts were determined by chromatographic analysis with an HPLC system equipped with a DAD detector. Since the HPLC method was calibrated using naringin as standard, their concentration is expressed as naringin equivalent (mg/g extract). 60 mg of sample were dissolved in 20 g of a mixture of water-ethanol (50/50, %*w*/*w*). The resulting solution was heated to 50 °C and vortexed for complete dissolution. Prior to being introduced into the autosampler vial, the solution was filtered with a 0.2 µm PTFE filter. The HPLC system used for the determination of the above flavonoids consists of a PerkinElmer Flexar Module equipped with a photodiode-array (PDA) detector, a series 200 autosampler, a series 200 peltier LC column oven, a series 200 LC pump, and a C18 Kinetex (particle size 5 µm, pore size 100 A, length and diameter 150 × 4.6 mm) column (Phenomenex, Torrance, CA, USA). Control of the HPLC system and data collection was accomplished on-line by a computer equipped with Chromera^®^ software (version 3.4.0.5712). Tests were performed in gradient mode with acetonitrile and water acidified by 0.1% of acetic acid (88%, *v*/*v*) as eluents using a flow rate of 1.4 mL/min, an injection volume of 3.0 µL, and a wavelength of 284 nm. The method was fully validated in terms of specificity, linearity, precision, accuracy, and quantitation limit (results are reported in the paragraph “Method S1. Method validation” of the Supplementary Materials).

### 2.7. Evaluation of Proanthocyanidin Presence in the Extracts

Proanthocyanidin presence was evaluated following two different methods. The first consisted of extract fractionation by gel permeation chromatography (GPC) according to the method developed by Gabetta et al. [26], with some modifications. GPC separations were performed at room temperature on a GE Healthcare Sephadex LH-20 (18–11 µm dry) column adapted for a Biotage Isolera^®^ flash chromatography system. Sephadex LH-20 (5.5 g) which had previously been suspended in ethanol/acetone (90/10, %*v*/*v*) was then packed into a column (7.0 × 2.0 cm i.d). 200 mg of each extract was dissolved in the initial mobile phases and loaded onto the pre-packed column. The separations were obtained by means of a binary linear gradient. The elution, under pressure, started using 10% of acetone and finished at 90% with 1000 mL as total volume of mobile phase at a constant flow rate of 5 mL/min. An aliquot of each of the obtained fractions was properly diluted in MeOH/HCOOH 100/0.1 (%*v*/*v*) and analyzed by ESI-MS (TSQ Quantum Ultra Triple Quadrupole, Thermo Finnigan, Milan, Italy) in negative ion mode. The ion source parameters were set as following: spray voltage −4.5 kV, sheath gas (nitrogen) 10 a.u., capillary temperature 275 °C, tube lens voltage 120 V. As reference, Leucoselect™ grape seed selected proanthocyanidins. from *V. vinifera* seeds was also fractionated and analyzed.

The second approach is based on the concept that tannins (such as proanthocyanidins) are able to precipitate proteins rich in proline residues (PRPs). The tannin effect was evaluated for bergamot extracts, Leucoselect™ and tannic acid as references by applying the method developed by Baron et al. [27]. Each compound/extract was dissolved in MeOH as stock solution and diluted properly in order to be added 1:10 in the final mixture with bradykinin (100 µM) in acetate buffer 50 mM. The mixtures were incubated for 10 min at 37 °C with different concentrations of tannic acid (0–200 µM), Leucoselect™ (0–2 mg/mL), BFPF (0–2 mg/mL), and BLPF (0–2 mg/mL), respectively. Samples were then centrifuged, and an aliquot of the supernatant diluted in H_2_O/CH_3_CN/HCOOH 70/30/0.1 (%*v*/*v*), added with the peptide LVNEVTEF (used as internal standard) and analyzed by ESI-MS (TSQ Quantum Ultra Triple Quadrupole, Thermo Finnigan, Milan, Italy).

### 2.8. Determination of Total Phenolic Content

The total phenolic content was measured by a modified Folin-Ciocalteu colorimetric method [28]. The extracts were prepared at a concentration of 100 µg/mL to obtain absorbance values within the linearity range of the standard curve (gallic acid 0.0–100.0 µg/mL). Aliquots of 100 µL of the Folin-Ciocalteu reagent (FCR) and 1 mL of distilled water were added to 200 µL of the extract. After 5 min standing, 700 µL of a 7% sodium carbonate aqueous solution was added to the samples. After 90 min at room temperature, absorbance was measured at 760 nm in a 96 well plate using a plate reader (BioTek’s PowerWave HT, Winooski, VT, USA). The total polyphenol content was expressed as mg of gallic acid equivalent per one gram of dry extract and reported as mean ± SD for five replicates.

### 2.9. Oxygen Radical Absorbing Capacity (ORAC) Assay

The antioxidant capacity of the extracts against oxygen radicals was tested with the ORAC assay following the protocol of Wang et al. with some minor modifications [29]. The extracts were prepared in water/ethanol (50/50, %*v*/*v*) at different concentrations (5–25 µg/mL) and 20 µM of trolox solution dissolved in the same solvents was used as reference. Aliquots of 250 µL of sample were mixed with 250 µL of a 2′,7′-dichloro-fluoresceine solution (500 nM) and 2 mL phosphate buffer (75 mM, pH 7.0). After radical activation at 37 °C for 10 min, 25 µL of 2,2′-azobis(2-methylpropionamidine) dihydrochloride (ABAP) solution (220 mM) was added to 475 µL of sample. Fluorescence was measured using an excitation wavelength of 485 nm and emission at 535 nm (Wallac Victor2 1420, Perkin-Elmer™ Life Science, Monza, Italy). The antioxidant activity was calculated by measuring the differences of AUC between the extracts and the blank, and the results expressed as micromoles of trolox equivalents per one gram of extract. Values are reported as mean ± SD of three replicates.

### 2.10. ABTS Radical Cation Decolorization Assay

The antioxidant activity against radical cation was also measured with the ABTS radical cation decolorization assay as reported by Re R. et al. [30]. ABTS radical cation (ABTS^•^) was produced by reacting ABTS stock solution (7 mM in water) with 2.45 mM potassium persulfate (final concentration) and allowing the mixture to stand in the dark at room temperature for 12–16 h before use. The solution was then diluted in ethanol to an absorbance of 0.70 (±0.02) at 734 nm. 180 µL of the solution so obtained was added to 20 µL of each sample analyzed in triplicate in a 96 well plate. A blank sample was also added to the plate. After 3 min at 30 °C the absorbance was measured at 734 nm using a plate reader (BioTek’s PowerWave HT, Winooski, VT, USA). The percentage of inhibition was calculated as expressed by Equation (2) and the results expressed as mean ± SD.

(2) $$\frac{{\mathrm{Abs}}_{\mathrm{blank}}{-\mathrm{Abs}}_{\mathrm{sample}}}{{\mathrm{Abs}}_{\mathrm{blank}}}\times 100$$

### 2.11. DPPH (2,2-Diphenyl-1-Picrylhydrazyl) Assay

The antioxidant capacity was also determined by the DPPH radical-scavenging method [31], with some modifications. An aliquot of 100 µL of the extract solution at different concentrations (1–25 µg/mL) was added to 750 µL of ethanol and 400 µL of acetate buffer (100 mM, pH 5.5), mixed and spiked with 250 µL of DPPH ethanolic solution (500 µM). After 90 min at room temperature and in the dark the absorbance at 515 nm was measured for each sample analyzed in triplicate with a UV reader Shimadzu™ UV 1900 (Shimadzu, Milano, Italia). The percentage of inhibition was calculated as expressed by Equation (3) and the results expressed as mean ± SD.

(3) $$\frac{{\mathrm{Abs}}_{\mathrm{blank}}{-\mathrm{Abs}}_{\mathrm{sample}}}{{\mathrm{Abs}}_{\mathrm{blank}}}\times 100$$

### 2.12. Cell Culture and Cell Stimulation

HEK293T (ATCC^®^, Manassas, VA, USA; accession number: CRL-3216™) and rat alveolar type I-like R3/1 clones were grown in Dulbecco modified Eagle medium (DMEM; Lonza, Verviers, Belgium) supplemented with 10% fetal bovine serum (FBS; Gibco, Gaithersburg, MD, USA), 1% glutamine (Lonza), and 1% penicillin/streptomycin (Gibco, Gaithersburg, MD, USA). R3/1-pLXSN cells (R3/1 control [32]) were used to generate, by lentiviral infection, a stable NF-κB signaling pathway reporter cell line (R3/1-NF-κB) using the lentivector pGreenFire-NF-κB-Puro (a kind gift from Dr. Darius Widera, University of Reading, UK; [33]) which drives the expression of both red firefly luciferase reporter and GFP in response to NF-κB activity.

For lentivirus production, 9 × 10^6^ 293T cells were seeded in a 15 cm plate and after 16 h the medium was changed to Iscove’s Modified Dulbecco’s Medium (IMDM; Lonza) containing 10% FBS, 1% penicillin/streptomycin and 1% glutamine 100 U/mL. Calcium phosphate precipitation method was used for transfection of lentiviral package vectors (7 µg pDM2-VSVG and 28 µg pCMV-ΔR8.91) and 32 µg pGreenFire-NF-κB-Puro. The medium was changed after 16 h to complete IMDM and butyrate sodium (1 µg/mL; Sigma, St Louis, MO, USA) and medium containing virus particles was collected 36 h later, centrifuged at 1500 rpm for 5 min, filtered through 0.22 µm filter (Merck, Germany) and centrifuged again at 20,000 rpm for 2 h at 4 °C. Viral particles were dissolved in 40 µL of sterile cold PBS and left in ice with moderate agitation for 30 min. Finally, virus aliquots were kept at −80 °C. For lentiviral transduction, R3/1 control cells (60,000) were seeded in a 12 well-plate and the next day fresh medium containing 6 µL (MOI 10) of virus particles was added for 24 h. Cells were then selected by treating cells with 1 µg/mL of puromycin for 4 days. Selected R3/1-NF-κB -cells were seeded at 3000 cells/well in a 96-wells plate for subsequent experiments.

To assess the anti-inflammatory activity, R3/1-NF-κB cells were pre-treated with the two extracts at different concentrations (10–250 µg/mL) for 18 h in complete medium, followed by a 6-h stimulation with 10 ng/mL IL-1α. In order to verify a possible direct interaction between the extract and IL-1α, the addition was performed with and without the removal of the medium containing the extract. Experiments were assayed by NF-κB luciferase activity, as described below.

### 2.13. NF-kB Luciferase Activity Assay

After treatment, R3/1-NF-κBcells were washed twice with cold PBS followed by a freeze-thaw cycle with reporter lysis buffer (purchased from Promega Corporation, Madison, WI, USA) for complete cell lysis. After the freeze-thaw cycle, 100 µL ONE-Glo™ Luciferase Assay Substrate (purchased from Promega Corporation, Madison, WI, USA) was directly added to the wells, followed by a luciferase measurement performed using a luminometer (Wallac Victor2 1420, Perkin-Elmer™ Life Science, Monza, Italy).

### 2.14. MTT Assay

The cell viability for the all the concentrations tested in the anti-inflammatory assay was verified by MTT assay on R3/1-NF-κB cells. After 18 h incubation with rosiglitazone (1–75 µM) and the extracts (10–250 µg/mL), 10 µL 5 mg/mL MTT reagent was added for 4 h. After medium removal, R3/1-NF-κB cells were lysed and MTT was solubilized by adding 100 µL of DMSO. The 96-well plate was shaken for 1 min and the absorbance at 490 nm was measured using a plate reader (BioTek’s PowerWave HT, Winooski, VT, USA). Cells incubated with DMSO (<0.1%) were used as a control for 100% cell proliferation.

### 2.15. Statistical Analysis

Biological experiments were performed with biological and technical replicates. Values are shown as mean ± SEM compared to untreated control cells. Statistical analysis was performed by using one-way ANOVA with Bonferroni correction. *p* < 0.05 was considered significant. Statistical analysis was performed with GraphPad Prism 6.02 for Windows, GraphPad Software, La Jolla, CA, USA, (www.graphpad.com) and OriginPro, version 2019, OriginalLab Corporation, Northampton, MA, USA.

## 3. Results

### 3.1. Targeted, Semi-Targeted and Untargeted Profiling by LC-HRMS

**Figure 1:**
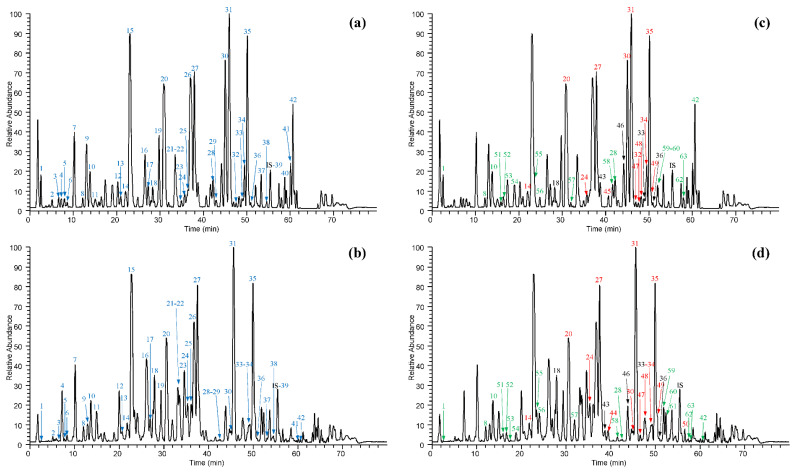
Total ion currents (TICs) of BFPF and BLPF extracts. In panels (**a**,**b**) the ions identified by the targeted approach are labelled in blue with a progressive number. In panels (**c**,**d**) the ions identified with the semi-targeted and untargeted approaches are labeled in red and green, respectively. (IS) Internal standard (Trolox); BFPF: panels (**a**,**c**); BLPF panels (**b**,**d**).

The analytical qualitative profile of the two extracts was carried out by using three different approaches: targeted, semi-targeted and untargeted. The first consisted of building a database of bergamot components already reported in the literature and containing 89 entries (Table S1 of Supplementary Materials). The identification of extract components in the db was made by matching the accurate masses, isotopic and fragmentation patterns. When possible, the identification of stereoisomers was performed on the basis of the elution order reported in previous papers which used RP chromatography as separation technique. The second approach (semi-targeted) was aimed at selectively identifying the most intense HMG derivatives which have interesting biological effects since they are involved in the cholesterol reducing activity of BPF; the approach consisted of mapping the characteristic ion losses of the HMG moiety (−62 Da, −102 Da and −144 Da): three different ion maps were generated from each of these losses and the common precursors were manually verified, thus giving a list of HMG-containing compounds. The putative identification was then accomplished by the accurate mass, isotopic and fragmentation patterns and by considering the match with molecular formula. The untargeted approach was focused on the identification of the most intense ions not identified using the previous methods: the elemental composition was calculated by the QualBrowser tool of Xcalibur as reported in the method section; the molecular formula was searched for using online databases such as MoNa and HMDB which generated a list of possible candidates whose structures had undergone in silico fragmentations. Simulated fragment ions were then compared to the experimental ones through the Peak Assignment tool of CFM-ID online software and the compounds putatively identified on the basis of the best fragmentation match [34,35]. A total number of 108 compounds were identified by using targeted, semi-targeted and untargeted profiling in bergamot leaf and fruit extracts, 100 of which are present in both the extracts thus demonstrating a good overlapping, at least from a qualitative point of view. Table 1 reports the retention times, accurate masses and fragmentation patterns of the metabolites identified by using the targeted approach; of the 61 metabolites identified, six were organic and phenolic acids, 24 flavones, 24 flavanones and seven limonoids. Most were present in both extracts, except for apigenin-7*-O-*rutinoside and neohesperidin*-O-*glucoside*-O-*HMG which were detected only in the leaf extract, while obacunone glucoside, limonin glucoside and obacunoic acid were detected only in BFPF. Some isomers were identified on the basis of their elution order as reported in previous studies which used RP chromatography as separation technique: for chrysoeriol/diosmetin isomers, chrysoeriol has always been reported as the first eluting isomer [18,19,21,25]; similarly, for rutinoside/neohesperidoside isomers the rutinoside is always the first eluting isomer followed by neohesperidoside [9,15,16,17,18,19,21,22,23,25]. Figure 1 (panels a and b) shows the total ion currents (TICs) of the two extracts where the peak ions identified by a targeted approach are labelled in blue by a time-dependent progressive number.

**Figure 2:**
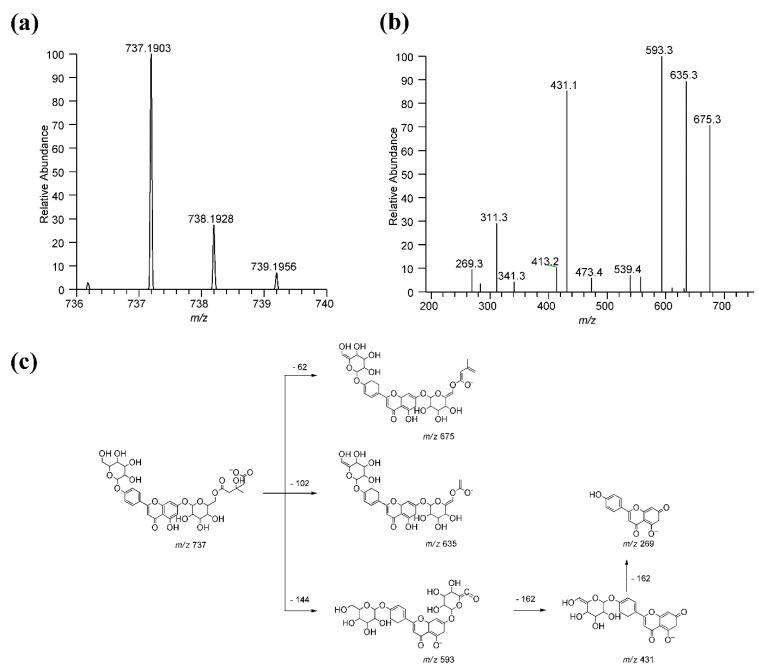
Identification of apigenin-di*-O-*glucoside*-O-*HMG by MS and MS/MS studies. Panel (**a**): full mass spectrum with accurate mass and isotopic pattern; panel (**b**): MS/MS spectrum; panel (**c**): proposed fragment tree MS/MS fragmentation.

Table 2 lists the 32 ions identified with the semi-targeted approach, including 11 compounds which were definitively confirmed since they had already been identified with the targeted approach. Of the remaining 21 compounds not yet identified in the bergamot plant, 17 were putatively assigned on the basis of the accurate mass, molecular formula, isotopic and fragmentation patterns while four HMG derivatives remain unknown. Thus, this approach allowed the detection of 20 HMG derivatives hitherto unreported in the literature, 17 found in both extracts, two only in leaf extract (luteolin*-O-*glucoside*-O-*HMG and an acetyl-glucosyl*-O-*HMG derivative), and one only in the fruit extract (bergamjuicin glucoside). Of these HMG derivatives, 6 out of 21 are flavone or flavanone di-glucosides, characterized by the loss of the HMG moiety (−144 Da) and by two neutral losses at −162 Da and −120 Da for the *O*-glucoside and C-glucoside, respectively; four are flavone mono glucosides, characterized by the neutral loss of the HMG moiety and of the sugar. The two flavanone rutinosides were recognized by the loss of the HMG moiety and by the loss of glucose and rhamnose (−146 Da). Bergamjuicin glucoside was characterized by the presence of an additional glucose moiety on the bergamjuicin structure. The compound 6-(beta-D-glucopyranosyloxy)-4-methoxy-5-benzofuranpropanoic acid*-O-*HMG was tentatively assigned on the basis of the identification of the HMG moiety and of the 6-(beta-d-glucopyranosyloxy)-4-methoxy-5-benzofuranpropanoic acid residue, this last annotated through the similarity search spectra of the MoNa database in the untargeted approach. Four identified HMG compounds characterized by the aglycones at *m/z* 315 (two isomers), 255 and 201 were not assigned. Table 3 lists the 26 compounds putatively identified by the untargeted method. MS and MS/MS spectra used for the putative identifications are collected in Figures S1–S26 of Supplementary Materials. Quinic acid has been previously identified in bergamot as an ester of sinapic acid [23], but not in a free form as in the current work. HMG-glucoside is here identified for the first time in bergamot and could derive from the hydrolysis of compounds bearing this moiety, such as melitidin. The two compounds 6-(beta-d-glucopyranosyloxy)-5-benzofuranpropanoic acid (also known as cnidioside A) and 6-(beta-d-glucopyranosyloxy)-4-methoxy-5-benzofuranpropanoic acid (also known as picraquassioside A) had also never been reported in bergamot although both of them were identified in an ethanolic extract of *Ruta graveolens* [36,37], which belongs to the Rutaceae family as does bergamot. The ion at *m/z* 265.1072 (compound 59) was tentatively assigned as 3-[2,4,5-trihydroxy-3-(3-methylbut-2-en-1-yl) phenyl] propanoic acid on the basis of its similarity to (3-[3,4-dihydroxy-5-(3-methylbut-2-en-1-yl) phenyl]-2-hydroxypropanoic acid), the latter was identified through the Compound identification tool of CFM-ID. The two compounds differ in the position of the hydroxy moiety, and the position on the assigned structure was made by considering that it can derive from compounds already present in bergamot and in particular by the opening of coumarin or by prenylation of the phenyl-propanoic acid moiety. Many of the compounds here putatively identified and not yet reported in the literature are glycosides of flavones and flavanones known to be present in bergamot, namely: neoeriocitrin*-O-*glucoside (or eriocitrin*-O-*glucoside), luteolin*-O-*neohesperidoside*-O-*glucoside (found in Citrus juices [38]), luteolin-C-glucoside*-O-*rhamnoside, luteolin*-O-*rutinoside, luteolin*-O-*neohesperidoside*-O-*rhamnoside, hesperetin-di-C-glucoside, naringenin-C-neohesperidoside*-O-*rhamnoside, apigenin*-O-*glucoside. Some other compounds are acetyl-derivatives of already known flavone and flavanone glycosides, such as luteolin*-O-*acetyl*-O-*neohesperidoside, naringenin-C-glucoside*-O-*acetyl-rhamnoside, diosmetin*-O-*acetyl-neohesperidoside (found in *Citrus aurantium* [39]). Figure 1 shows the TIC traces of BFPF and BLPF where peak ions identified by the semi-targeted and untargeted methods are labelled in red and green, respectively. Peaks reporting more than one number indicate the co-elution of more than one ion. For each identified compound, the mass spectrum together with the isotopic pattern and the relative MS/MS spectrum were analyzed and where possible, the fragmentation tree generated. As an example, Figure 2 shows the MS and MS/MS results relative to apigenin-di*-O-*glucoside.

### 3.2. Semi-Quantitative Analysis of the Identified Metabolites

**Figure 3:**
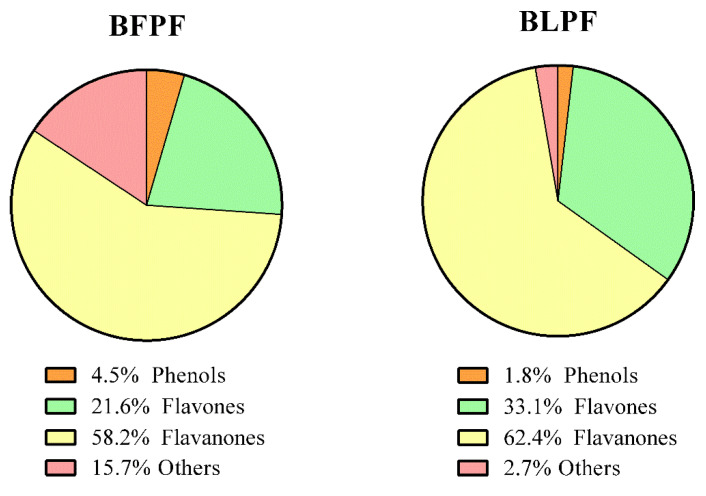
Percentage composition calculated on the base of peak areas of BLPF and BFPF. Flavones are in green, flavanones in yellow, phenols in orange and organic acids in red.

**Figure 4:**
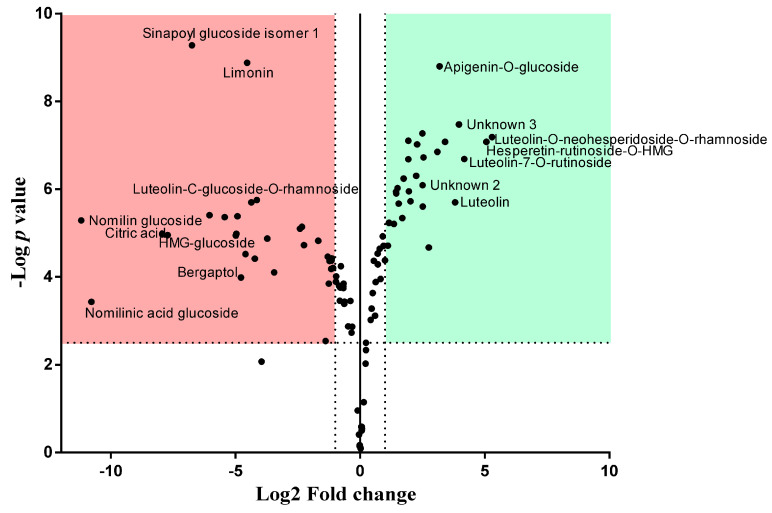
Volcano plot showing the semi-quantitative difference for each metabolite present in the two extracts. Those compounds significantly higher in BFPF are in red, while those significantly higher in BLPF are in green. The most statistically different compounds are named.

The identified metabolites are clustered into three main classes: phenols, polyphenols (flavones and flavanones) and “others”, which include organic acids, limonoids, coumarins and unknowns. Figure 3 shows the relative content of each class calculated on the basis of the peak areas: BLPF comprises 1.8% phenols, 95.5% polyphenols (33.1% flavones and 64.2% flavanones) and 2.7% others; BFPF contains a higher percentage of phenols (4.5%) and others (15.7%), while polyphenols are 79.8% (21.6% flavones and 58.2% flavanones). Table 4 reports the fold change (log2 value) of the relative abundance of the 100 common compounds in BFPF versus BLPF and the statistical significance (−Log *p* value). 41 compounds were found to have a similar relative abundance (log2 fold change between −1 and 1), 31 with a relative abundance higher in BFPF (red region, log2 fold change < −1) and 28 lower (green region, log2 fold change > 1) in respect to leaf extract. Figure 4 shows the data in a graphical form (Volcano plot).

**Figure 5:**
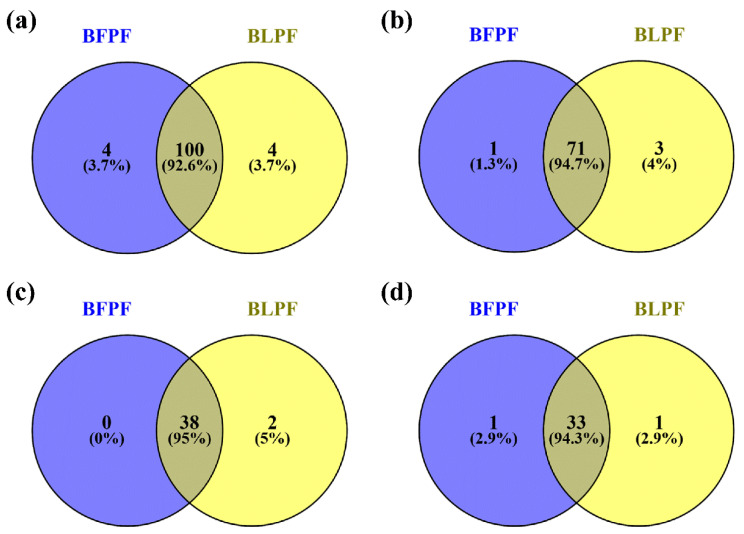
Venn diagrams showing the qualitative comparison between the two extracts: (**a**) of all the identified compounds, 100 (92.6%) are in common between the two extracts; (**b**) 94.7% of the identified polyphenols are in common; (**c**) all the flavones identified in BFPF are also present in BLPF which contains 2 more flavones; (**d**) of the identified flavanones, 33 (94.3%) are present in both the extract.

Figure 5 summarizes the qualitative differences between the two extracts as Venn diagrams: 92.6% of the compounds are in common (100), four compounds were found only in BFPF and 4 only in the leaf extract (Figure 5a). Regarding polyphenols, 71 (94.7%) are present in both the extracts (Figure 5b) and, among these, all the 38 flavones identified in BFPF are also present in BLPF which contains two more (Figure 5c), while 94.3% of the identified flavanones are in common (Figure 5d). Overall, we can say that the polyphenol pattern does not substantially differ.

### 3.3. Quantitative Analysis

The quantitative analysis of the major flavonoids (neoeriocitrin, naringin, neohesperidin, melitidin, and brutieridin) present in bergamot extracts was performed by HPLC-UV. Naringin was used as standard and the results are expressed as naringin equivalent (mg/g extract) (Table 5).

### 3.4. Proanthocyanidin Analysis

Proanthocyanidin were not detected in either the bergamot extracts by using both the methods reported in the method section. Results are reported in Figures S27–S30 of the Supplementary Materials.

### 3.5. Total Phenolic Content and Antioxidant Activity

The mean values (±SD) of the total phenolic content and the antioxidant activity measured with the ORAC, DPPH and ABTS assays are reported in Table 6, ascorbic acid values obtained from the literature are also reported as a reference [40,41,42]. The total phenolic content was higher in the leaf extract in respect to BFPF (by almost 38%) and the difference was statistically different (*p* = 0.0328). Regarding the antioxidant activity, the ORAC value was higher in BLPF in respect to BFPF (a higher value indicates a higher antioxidant activity) but not statistically different (*p* = 0.331). When the ORAC values were normalized in respect to the polyphenol content, the values almost overlapped (*p* = 0.802). The antioxidant activity tested with DPPH was found to be higher for BLPF, showing a potency almost doubled in respect to BFPF and statistically different (*p* < 0.0001). The difference, although reduced, remained significant when the values were normalized in respect to the polyphenol content (*p* = 0.0232). The higher antioxidant activity of BLPF (almost double) was confirmed by the ABTS method (*p* = 0.0012) but was not significantly different when normalized in respect to the polyphenol content (*p* = 0.0951).

### 3.6. Anti-Inflammatory Activity

**Figure 6:**
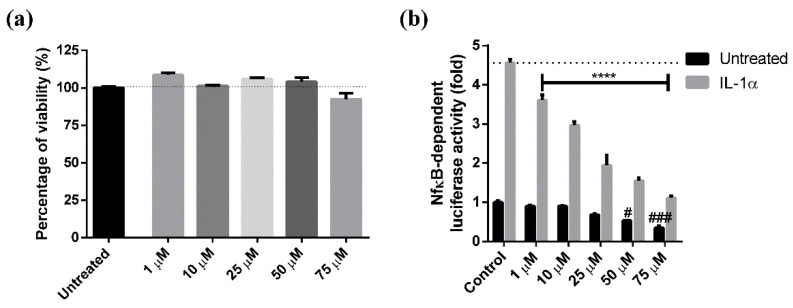
Dose-dependent effect of rosiglitazone used as reference active compound on R3/1-NF-κB cells (**a**) viability (MTT assay) and (**b**) IL-1α induced NF-κB activation. Data are reported as mean ± SEM. The statistical significance difference of each concentration in respect to the control was analyzed by ONE-WAY analysis followed by the Bonferroni post-test. For the untreated series: ^#^ *p* < 0.05, ^###^ *p* < 0.001. For IL-1α treated samples: **** *p* < 0.0001.

**Figure 7:**
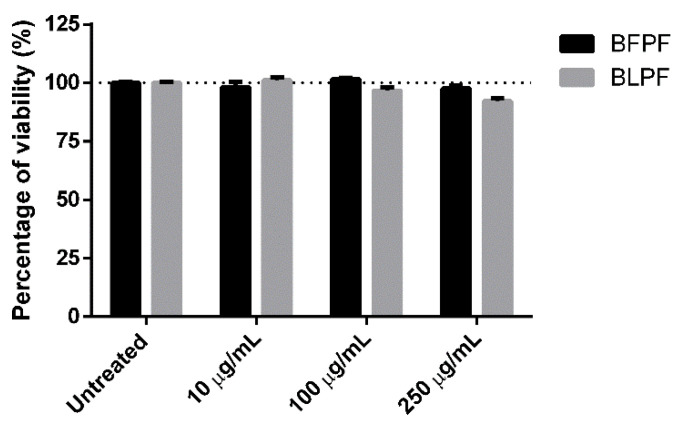
Dose-dependent effect of BFPF and BLPF on cell viability as measured by MTT on R3/1-NF-κB cells. Data are shown as mean ± SEM.

**Figure 8:**
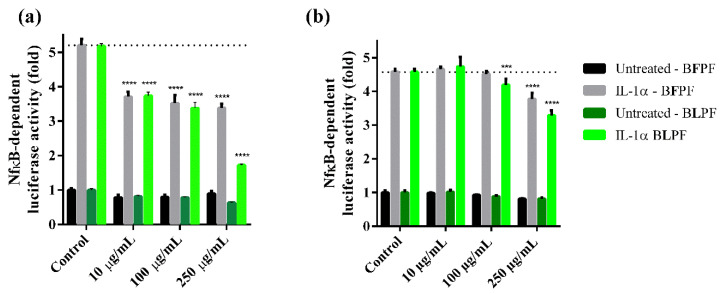
Dose-dependent effect of BFPF and BLPF on IL-1α induced NF-κB activation. R3/1-NF-κB cells were incubated for 18 h with different concentrations of BLPF (green) or BFPF (grey), followed by a 6 h stimulation with IL-1α, without the removal of medium containing the extract (**a**) or with the removal (**b**). Then NF-κB dependent luciferase activity was measured. Data are reported as mean ± SEM. The statistical significance difference of each concentration of BFPF and BLPF in respect to the control was analyzed by ONE-WAY analysis followed by the Bonferroni post-test. *** *p* < 0.001 and **** *p* < 0.0001.

To evaluate and compare the anti-inflammatory effect of the two extracts, R3/1 control cells were stably transduced with an NF-κB-driven reporter encoding for luciferase to generate R3/1-NF-κB cells. When stimulated with IL-1α, a potent inducer of the pro-inflammatory pathway through NF-κB, transduced R3/1-NF-κB cells reported a luminescence signal increased more than 5-fold in respect to control cells (Figure 6). The cell model was validated by using rosiglitazone which is a well-known anti-inflammatory agent via NF-κB inhibition (Figure 6). The anti-inflammatory activity of the two extracts was tested by using two different protocols: in one case the pro-inflammatory agent IL-1α was incubated in the presence of the extract while in the second case it was added after removing the extract. Both the extracts were found not to affect the cell viability in the concentration range 10–250 µg/mL as determined by the MTT assay (Figure 7). A dose-dependent anti-inflammatory effect was observed for both the extracts. As expected, the anti-inflammatory activity was higher when IL-1α was incubated in the presence of the extract: in such a condition both the extracts were found to be already active at 10 µg/mL while when the extracts were removed, the activity was significant in a concentration range between 100 and 250 µg/mL for BLPF and at 250 µg/mL for BFPF (Figure 8). BLPF was found to be significantly more effective in respect to BFPF at 250 µg/mL when the extract was removed and at all the concentrations tested in the second cell experiment.

## 4. Discussion

By using targeted, semi-targeted and untargeted approaches, the qualitative and semi-quantitative profiling of the enriched polyphenol fraction obtained from bergamot leaves and fruit was compared. Overall, the qualitative composition is quite overlapping since 71 polyphenolic components were found in both the extracts, accounting for 98.7% and 96% of the total polyphenols present in leaf and fruit, respectively. Some polyphenols were only found in fruit extract and others only in leaf extract, accounting for 1.3 and 4%, respectively. Regarding the quantitative aspect, it was possible to compare by a semi-quantitative approach the polyphenol content of the two extracts due to the fact that the same method for polyphenol enrichment was used. The total polyphenol content was 38% higher in leaf in respect to fruit, suggesting a higher content of polyphenols in the starting raw material. The non-polyphenolic constituents were also addressed as summarized in Table 1 (analytes are classified as polyphenols, phenols and non-phenolics). The higher polyphenolic content in the leaf correlates to the higher relative content of the non-polyphenolic constituents in BFPF in respect to BLPF. Organic acids and non-phenolic constituents (others) account in BFPF for 4.5% and 15.7%, respectively, (sum of 20.2%) as opposed to 1.8% and 2.7% in leaf (sum of 4.5%). By contrast, polyphenols, and in particular flavanones and flavones, are lower in BFPF in respect to BLPF, being 21.6% and 58.2% (79,8%) as opposed to 33.1% and 62.4% (sum of 95.5%), respectively. Hence the polyphenolic qualitative composition is quite overlapping in the two extracts while the relative content is higher in leaf and the non-polyphenolic amount is higher in the fruit. Besides comparing the extracts, and profiling for the first time the bergamot leaf composition, in the present study compounds not previously reported in bergamot were identified in BFPF, whose biological and pharmacological activity is emerging. In particular, by using a semi-targeted approach aimed at identifying the HMG-derivatives, 21 more components carrying the HMG moiety were identified. These compounds deserve further investigation since the cholesterol lowering effect of bergamot polyphenol extract has been attributed to the modulation of the key enzyme 3-hydroxy-3-methylglutaryl-CoA reductase (HMGR). In particular, computational studies have suggested that HMG derivatives have the ability to replace HMG-CoA, the endogenous substrate of HMG-CoA reductase [11]. Such a mechanism has been proposed to explain the ability of BPF to inhibit cholesterol synthesis and in particular to act as a cholesterol-lowering food supplement as demonstrated in intervention studies performed on metabolic syndrome patients. The identification of other HMG derivatives further improves our understanding of bergamot’s cholesterol lipid lowering effect and would permit the investigation of the effect of these identified molecules on HMGR by using different approaches such as molecular docking which is currently applied in our lab. HMG derivatives were also identified in leaves and the profile is quite overlapping, but two more HMG components were detected, for which an annotation was possible only for luteolin*-O-*glucoside*-O-*HMG, while for the compounds at *m/z* 549.1600 we were not able to give a hypothesis of characterization. Bergamjuicin glucoside was instead putatively identified only in BFPF. By using an untargeted approach, we then searched for other compounds in BPF in respect to those reported in the literature. We found 26 more compounds as shown in Table 3. The higher polyphenol content in leaves in respect to the fruit partially explains the higher antioxidant activity as determined by three different methods, ORAC, DPPH and ABTS. In particular, the antioxidant activity of BLPF was found to be higher than that of fruit in all the three methods and accounts for 34%, 52% and 48%, respectively. The difference was found to be significant for DPPH and ABTS but not for ORAC, probably due to the limited precision of this method (CV%, 26%) in respect to the two others. By normalizing the antioxidant activity in respect to the polyphenol content, the differences in antioxidant activities between the two extracts greatly reduced (7%, 34%, 29% for the ORAC, DPPH and ABTS) and were found significantly different only for the ABTS method. The data suggest that the higher antioxidant activity found in leaves in respect to the BPF is due to their different polyphenol content and not to a different composition and this is confirmed because they become similar when normalized on the basis of the polyphenol content. Compared to known antioxidants the two extracts showed a good antioxidant activity: ascorbic acid is reported to have 4318 TE/g as ORAC value [40], a IC_50_ mean value of 41.2 µg/mL for the DPPH assay [41], and a IC_50_ value of 28.2 µg/mL for the ABTS assay [42]. Finally, we evaluated the effects of leaf and fruit on cell inflammation. BFPF has been reported to possess a significant anti-inflammatory activity in both in vitro and in vivo models through both an antioxidant effect and by a mechanism involving the inhibition of NF-κB activation. Such anti-inflammatory activity has been addressed to flavanone and flavone glycosides, as well as to their aglycones, including naringin and hesperidin, diosmetin, apigenin, and luteolin glycosides, which are contained in both leaf and fruit extracts. The two extracts were both found effective when incubated and pre-incubated with the inflammatory stimulus although they are more effective in the former condition. In both the experiments leaf extract was significantly more effective than the fruit extract in accordance with its higher antioxidant activity and higher polyphenol content. Hence the data confirm that the anti-inflammatory activity is due to the polyphenol fraction which is higher in leaves in respect to BFPF.

In conclusion, the comparison of the qualitative and quantitative profile of polyphenols as well as of the antioxidant and anti-inflammatory activity of bergamot leaf and fruit well indicates that leaf is a valid source of bergamot polyphenol extraction and even a richer source of polyphenol in respect to the fruit. The similar qualitative pattern of polyphenol components and of HMG derivatives suggest that leaf extract should possess a similar pharmacological activity to that of BFPF which needs to be confirmed in animal studies.

## Figures and Tables

**Table 1:** Compounds identified in both BFPF and BLPF with the targeted approach. * only present in the BLPF. ** only present in BFPF.

Peak	Compound	RT (min)	*m/z*	MS/MS Ion Fragments	Molecular Formula	
	**Non-phenolics**					
	*Organic Acids*					
1	Citric acid	2.5	191.0202	111-147	C_6_H_8_O_7_	1.571
	*Limonoids*					
28	Nomilin glucoside	41.8	693.2748	427-471-565-607-633-651	C_34_H_46_O_15_	−0.517
30	Nomilinic acid glucoside	45.0	711.2850	607-651	C_34_H_48_O_16_	−0.862
33	Obacunone glucoside **	48.9	633.2535	331-359-427-589	C_32_H_42_O_13_	−0.668
35	Limonin glucoside **	49.8	649.2502	341-385-443-461-587-605	C_32_H_42_O_14_	1.118
40	Obacunoic acid **	58.8	471.2017	203-245-307-325-351-409-427	C_26_H_32_O_8_	0.346
41	Limonin	60.1	469.1872	229-278-283-306-321-381	C_26_H_30_O_8_	1.506
42	Nomilinic acid	60.6	531.2220	427-471-489	C_28_H_36_O_10_	−0.474
	**Phenols**					
	*Phenolic Acids*					
2	Feruloyl glucoside isomer 1	5.2	355.1035	193	C_16_H_20_O_9_	3.271
3	Sinapoyl glucoside isomer 1	6.4	385.1135	223	C_17_H_22_O_10_	0.567
5	Feruloyl glucoside isomer 2	8.0	355.1033	193	C_16_H_20_O_9_	0.941
6	Sinapoyl glucoside isomer 2	8.6	385.1136	223	C_17_H_22_O_10_	0.697
9	2-Hydroxy-4-methoxyhydrocinnamoyl-2*-O-*glucoside	13.0	357.1185	151-177-195	C_16_H_22_O_9_	0.491
	**Polyphenols**					
	*Flavones*					
4	Luteolin-6,8-di-C-glucoside	7.2	609.1443	368-399-429-471-489-519	C_27_H_30_O_16_	−0.711
7	Apigenin-6,8-di-C-glucoside	10.2	593.1495	353-383-473-503	C_27_H_30_O_15_	−0.596
8	Chrysoeriol-6,8-di-C-glucoside	12.1	623.1600	312-383-413-503-533	C_28_H_32_O_16_	−0.661
10	Diosmetin-6,8-di-C-glucoside	13.7	623.1603	312-383-413-503-533	C_28_H_32_O_16_	−0.361
11	Luteolin-7*-O-*glucoside	15.1	447.0924	285	C_21_H_20_O_11_	0.212
13	Apigenin-8-C-glucoside	20.7	431.0983	269-283-311-341	C_21_H_20_O_10_	1.027
14	Apigenin-6-C-glucoside	21.8	431.0979	269-283-311-341	C_21_H_20_O_10_	0.627
	Chrysoeriol-8-C-glucoside	25.9	461.1084	341-371	C_22_H_22_O_11_	0.562
16	Luteolin-7*-O-*neohesperidoside	26.5	593.1496	285-447	C_27_H_30_O_15_	−0.496
17	Diosmetin-8-C-glucoside	27.0	461.1090	341-371	C_22_H_22_O_11_	1.162
20	Apigenin-7*-O-*rutinoside *	30.4	577.1575	269	C_27_H_30_O_14_	2.318
21	Apigenin-7*-O-*neohesperidoside	33.2	577.1557	269	C_27_H_30_O_14_	0.518
23	Chrysoeriol-7*-O-*glucoside	35.0	461.1079	284-299	C_22_H_22_O_11_	0.062
24	Diosmetin-7*-O-*glucoside	35.8	461.1082	284-299	C_22_H_22_O_11_	0.362
25	Chrysoeriol-7*-O-*neohesperidoside	36.2	607.1655	284-299	C_28_H_32_O_15_	−0.247
25	Demethoxycentaureidin-7*-O-*glucoside	36.3	491.1199	314-329-371	C_23_H_24_O_12_	1.498
27	Diosmetin-7*-O-*neohesperidoside	37.9	607.1655	284-299	C_28_H_32_O_15_	−0.247
32	Apigenin-7*-O-*neohesperidoside*-O-*HMG	47.2	721.1959	577-619-659	C_33_H_38_O_18_	−1.541
34	Luteolin	49.1	285.0400	151-175-191-199-217-241-243	C_15_H_10_O_6_	0.636
34	Diosmetin-7*-O-*neohesperidoside*-O-*HMG	49.1	751.2095	299-461-607-649-689	C_34_H_40_O_19_	1.495
36	Demethoxycentaureidin-7*-O-*glucoside-HMG	50.9	635.1604	314-329-491-533	C_29_H_32_O_16_	−0.261
38	Apigenin	54.5	269.0450	149-175-225	C_15_H_10_O_5_	0.550
39	Chrysoeriol	55.5	299.0557	256-271-284	C_16_H_12_O_6_	0.685
39	Diosmetin	55.7	299.0558	256-284	C_16_H_12_O_6_	0.785
	*Flavanones*					
	Naringin-glucoside	14.7	741.2249	271-459-479	C_33_H_42_O_19_	1.245
12	Eriodictyol 7*-O-*rutinoside (Eriocitrin)	20.0	595.1657	287	C_27_H_32_O_15_	−0.047
13	Eriodictyol-7*-O-*glucoside	20.5	449.1080	287	C_21_H_22_O_11_	0.162
15	Eriodictyol 7*-O-*neohesperidoside (Neoeriocitrin)	22.9	595.1651	287-449	C_27_H_32_O_15_	−0.647
18	Naringenin 7*-O-*rutinoside (Narirutin)	27.6	579.1713	271	C_27_H_32_O_14_	0.468
18	Neoeriocitrin-glucoside*-O-*HMG	28.0	901.2591	287-595-637-377-739-757-799-839	C_39_H_50_O_24_	−1.728
19	Naringenin-7*-O-*glucoside (Prunasin)	29.4	433.1133	271	C_21_H_22_O_10_	0.377
19	Bergamjuicin (Melitidin-glucoside)	29.5	885.2640	271-459-579-621-661-723-741-783-823	C_39_H_50_O_23_	−1.914
20	Neohesperidin-glucoside*-O-*HMG *	30.3	915.2730	301-609-651-691-771-813-853	C_40_H_52_O_24_	−3.479
20	Naringenin 7*-O-*neohesperidoside (Naringin)	30.5	579.1705	271	C_27_H_32_O_14_	−0.332
	Hesperetin*-O-*glucoside isomer 1	32.4	463.1241	301	C_22_H_24_O_11_	0.612
22	Hesperetin 7*-O-*rutinoside (Hesperidin)	33.6	609.1816	301-489	C_28_H_34_O_15_	0.203
25	Hesperetin*-O-*glucoside isomer 2	36.3	463.1239	301	C_22_H_24_O_11_	0.412
26	Hesperetin 7*-O-*neohesperidoside (Neohesperidin)	36.7	609.1809	301-447-489	C_28_H_34_O_15_	−0.497
27	Neoeriocitrin*-O-*HMG	37.6	739.2067	287-433-595-637-677	C_33_H_40_O_19_	−1.305
29	Eriodictyol	42.6	287.0560	135-151	C_15_H_12_O_6_	0.985
30	Naringenin 7*-O-*glucoside*-O-*HMG	44.8	577.1554	271-433-475-515	C_27_H_30_O_14_	0.378
31	Melitidin (Naringin*-O-*HMG)	45.7	723.2148	579-621-661	C_33_H_40_O_18_	1.709
32	Hesperetin*-O-*glucoside*-O-*HMG isomer 1	47.1	607.1666	301-463-505-545	C_28_H_32_O_15_	0.853
34	Hesperetin*-O-*glucoside*-O-*HMG isomer 2	49.8	607.1651	301-463-505-545	C_28_H_32_O_15_	−0.647
35	Brutieridin (Neohesperidin*-O-*HMG)	50.0	753.2223	609-651-691	C_34_H_42_O_19_	−1.355
37	Naringenin	53.2	271.0608	107-119-151-165-177-227	C_15_H_12_O_5_	0.700
38	Isosakuranetin-7*-O-*neohesperidoside*-O-*HMG	54.4	737.2266	285-411-593-635-675	C_34_H_42_O_18_	2.596
39	Hesperetin	55.3	301.0714	151-174-199-242-258-268	C_16_H_14_O_6_	0.735

**Table 2:** Compounds identified * and putatively identified ** with the semi-targeted method divided for flavonoid classes. In bold the compounds only present in BLPF, in italics those only present in BFPF.

Peak	Identification/Putative Identification	RT (min)	*m/z*	MS/MS Ion Fragments	Molecular Formula	Δppm
	**Polyphenols**					
	*Flavones*					
14	Apigenin-di*-O-*glucoside*-O-*HMG **	21.4	737.1903	269-431-593-635-675	C_33_H_38_O_19_	−2.774
	Diosmetin-di-C-glucoside*-O-*HMG **	25.6	767.1998	299-341-461-605-647	C_34_H_40_O_20_	−4.066
	Chrysoeriol-di*-O-*glucoside*-O-*HMG **	28.6	767.2006	299-461-503-605	C_34_H_40_O_20_	−3.024
	Diosmetin-di*-O-*glucoside*-O-*HMG **	29.7	767.1998	299-461-503-605	C_34_H_40_O_20_	−4.066
	Luteolin*-O-*glucoside*-O-*HMG **	37.4	591.1353	285-447-489-529	C_27_H_28_O_15_	1.444
44	Luteolin*-O-*neohesperidoside*-O-*HMG **	39.5	737.1934	285-447-593-635-675	C_33_H_38_O_19_	1.417
47	Apigenin*-O-*glucoside*-O-*HMG **	46.6	575.1403	269-431-473-513	C_27_H_28_O_14_	1.336
32	Apigenin-7*-O-*neohesperidoside*-O-*HMG *	47.2	721.1967	577-619-659	C_33_H_38_O_18_	−1.027
33	Chrysoeriol*-O-*glucoside*-O-*HMG **	48.8	605.1508	299-461-503-543	C_28_H_30_O_15_	1.163
34	Diosmetin-7*-O-*neohesperidoside*-O-*HMG *	49.1	751.2056	299-461-607-649-689	C_34_H_40_O_19_	−3.202
36	Demethoxycentaureidin-7*-O-*glucoside*-O-*HMG *	50.9	635.1604	314-329-491-533	C_29_H_32_O_16_	−0.261
49	Diosmetin*-O-*glucoside*-O-*HMG **	50.5	605.1505	299-461-503-543	C_28_H_30_O_15_	0.667
	*Flavanones*					
	Eriodictyol-di*-O-*glucoside*-O-*HMG **	24.9	755.2003	287-449-491-531-593	C_33_H_40_O_20_	−3.469
18	Neoeriocitrin*-O-*glucoside*-O-*HMG *	28.0	901.2591	287-595-637-377-739-757-799-839	C_39_H_50_O_24_	−1.918
20	Neohesperidin*-O-*glucoside*-O-*HMG *	30.3	915.2731	301-609-651-691-771-813-853	C_40_H_52_O_24_	−3.724
	Eriodictyol*-O-*glucoside*-O-*HMG **	35.4	593.1495	287-449-491-531	C_27_H_30_O_15_	−0.596
24	Eriocitrin*-O-*HMG **	35.8	739.2052	287-433-595-637-677	C_33_H_40_O_19_	−2.805
27	Neoeriocitrin*-O-*HMG *	37.6	739.2067	287-433-595-637-677	C_33_H_40_O_19_	−1.766
45	*Bergamjuicin glucoside*	40.6	1047.3167	741-885-903-945-985	C_45_H_60_O_28_	−1.993
46	Naringenin*-O-*rutinoside*-O-*HMG **	43.9	723.2142	271-417-579-621-661	C_33_H_40_O_1_	1.534
30	Naringenin 7*-O-*glucoside*-O-*HMG *	44.8	577.1554	271-433-475-515	C_27_H_30_O_14_	0.378
31	Melitidin (Naringin*-O-*HMG) *	45.7	723.2148	579-621-661	C_33_H_40_O_1_	2.364
32	Hesperetin*-O-*glucoside*-O-*HMG isomer 1 *	47.1	607.1666	301-463-505-545	C_28_H_31_O_15_	1.406
48	Hesperetin*-O-*rutinoside*-O-*HMG **	47.7	753.2221	301-609-651-691	C_34_H_42_O_19_	−2.065
33	Hesperetin-di*-O-*glucoside*-O-*HMG **	48.9	769.2156	301-463-625-667-707	C_34_H_42_O_20_	−3.861
34	Hesperetin*-O-*glucoside*-O-*HMG isomer 2 *	49.8	607.1647	301-463-505-545	C_28_H_31_O_15_	−1.724
35	Brutieridin (Neohesperidin*-O-*HMG) *	50.0	753.2223	609-651-691	C_34_H_42_O_19_	−1.799
	**Non-phenolics**					
43	6-(beta-D-glucopyranosyloxy)-4-methoxy-5-benzofuranpropanoic acid*-O-*HMG **	38.4	541.1556	191-217-235-397-439-479	C_24_H_30_O_14_	0.773
	**Unknown**					
	315-glucoside*-O-*HMG (1)	39.0	621.1450	300-315-477-519-559-579	C_28_H_30_O_16_	−0.018
	315-glucoside*-O-*HMG (2)	46.2	621.1451	300-315-477-519-559-579	C_28_H_30_O_16_	0.143
	255-C-glucoside*-O-*rhamnoside*-O-*HMG	54.1	707.2159	255-357-401-563-605-645	C_33_H_40_O_17_	−3.218
50	**201-acetyl-glucosyl*-O-*HMG**	56.7	549.1600	201-243-405-447	C_26_H_30_O_13_	−0.487

**Table 3:** Compounds putatively identified with the untargeted approach.

Peak	Putative Identification	RT (min)	*m/z*	MS/MS Ion Fragments	Molecular Formula	Δppm
	**Non-phenolics**					
1	Quinic acid	2.2	191.0564	133-147	C_7_H_12_O_6_	1.385
1	HMG-glucoside	2.5	323.0974	161-179	C_12_H_20_O_10_	0.393
54	6-(beta-D-glucopyranosyloxy)-5-benzofuranpropanoic acid	19.1	367.1028	161-205	C_17_H_20_O_9_	0.481
55	6-(beta-D-glucopyranosyloxy)-4-methoxy-5-benzofuranpropanoic acid	23.3	397.1135	176-191-217-235	C_18_H_22_O_10_	0.567
58	6-hydroxy-4-methoxy-5-benzofuranpropanoic acid	41.5	235.0611	176-191	C_12_H_12_O_5_	1.010
28	Bergaptol	42.1	201.0194	157	C_11_H_6_O_4_	1.165
62	Deacetylnomilinic acid	57.5	489.2125	325-333-411	C_26_H_34_O_9_	0.611
63	Limonoate A-ring lactone	58.0	487.1953	383-427	C_26_H_32_O_9_	−1.968
42	Deacetylnomilin	60.5	471.2015	307-325-409	C_26_H_32_O_8_	0.330
	**Phenols**					
10	p-Coumaric acid	13.8	163.0406	119	C_9_H_8_O_3_	1.629
59	3-[2,4,5-trihydroxy-3-(3-methylbut-2-en-1-yl)phenyl]propanoic acid	51.9	265.1072	87-151-163-177-185-203-221	C_14_H_18_O_5_	0.100
	**Polyphenols**					
8	Neoeriocitrin*-O-*glucoside/eriocitrin*-O-*glucoside	11.9	757.2160	287-449-595	C_33_H_42_O_20_	−3.394
51	Luteolin*-O-*neohesperidoside*-O-*glucoside	15.5	755.2001	285-447-593	C_33_H_40_O_20_	−3.694
53	Luteolin-C-glucoside*-O-*rhamnoside	17.5	593.1502	285-447-473	C_27_H_30_O_15_	0.114
55	Luteolin*-O-*rutinoside	23.5	593.1501	285	C_27_H_30_O_15_	−1.860
56	Luteolin*-O-*neohesperidoside*-O-*rhamnoside	24.1	739.2050	285-593	C_33_H_40_O_19_	−4.065
18	Hesperetin-di-C-glucoside	27.4	625.1756	301-343-463-505	C_28_H_34_O_16_	−2.892
18	Naringenin-C-glucoside-di*-O-*rhamnoside	27.4	725.2299	271-459-605	C_33_H_42_O_18_	1.599
57	Apigenin*-O-*glucoside	31.9	431.0976	269	C_21_H_20_O_10_	0.327
43	Luteolin*-O-*acetyl*-O-*neohesperidoside	38.4	635.1610	285-327-489-593	C_29_H_32_O_16_	0.339
46	Naringenin-C-glucoside*-O-*acetyl-rhamnoside	43.9	621.1812	271-313-459-501-579	C_29_H_34_O_15_	−0.316
33	Diosmetin*-O-*acetyl-neohesperidoside	48.8	649.1771	284-299-607	C_30_H_34_O_16_	0.749
	**Unknown**					
52	Unknown 1	15.8	611.1617	287-329-373-449-475-491	C_27_H_32_O_16_	1.700
36	255-C-glucoside*-O-*rhamnoside	51.3	563.1769	255-279-297-401-443	C_27_H_32_O_13_	1.745
60	Unknown 2	52.4	417.0819	129-161-173-189-251-277-295	C_20_H_18_O_10_	0.227
61	Unknown 3	53.7	417.0816	129-161-173-189-251-277-295	C_20_H_18_O_10_	0.017

**Table 4:** Log2 Fold change and −Log *p* values for the 100 common compounds of the two extracts.

Compound	Log2 Fold Change	−Log *p* Value	Compound	Log_2_ Fold Change	−Log *p* Value	Compound	Log2 Fold Change	−Log *p* Value
Log2 Fold Change < −1			−1 < Log2 Fold Change < 1			Log2 Fold Change > 1		
Nomilin glucoside	−11.1948	5.287815	Feruloyl acid glucoside isomer 2		3.891729	Naringenin-7*-O-*glucoside*-O-*HMG	1.10573	4.71474
Nomilinic acid glucoside	−10.7842	3.435279	Apigenin-7*-O-*neohesperidoside*-O-*HMG	-0.96767	4.013113	Diosmetin	1.154867	5.2347
Citric acid	−7.94861	4.9829	Hesperetin*-O-*glucoside isomer 1	-0.8536	3.801555	Luteolin-7*-O-*neohesperidoside	1.352863	5.210871
HMG-glucoside	−7.73033	4.955343	Diosmetin*-O-*glucoside*-O-*HMG	-0.8239	3.459949	Neoeriocitrin*-O-*glucoside*-O-*HMG	1.439913	5.944729
Sinapoyl glucoside isomer 1	−6.75049	9.282999	Diosmetin-7*-O-*neohesperidoside*-O-*HMG	-0.80672	3.759166	Chrysoeriol-7*-O-*glucoside	1.447237	5.907507
Limonoate A-ring lactone	−6.05197	5.41094	Diosmetin-di-C-glucoside*-O-*HMG	-0.77604	4.249155	Apigenin*-O-*glucoside*-O-*HMG	1.493765	6.026196
6-hydroxy-4-methoxy-5-benzofuranpropanoic acid	−5.44157	5.363352	Naringenin-C-glucoside*-O-*acetyl-rhamnoside	-0.67462	3.74815	Chrysoeriol	1.539391	5.672604
p-Coumaric acid	−4.9956	4.94969	Apigenin-8-C-glucoside	-0.67358	3.790493	315-glucoside*-O-*HMG (1)	1.69047	5.343194
Hesperetin	−4.97399	4.984615	Chrysoeriol-8-C-glucoside	-0.66776	3.850652	Hesperetin-di-C-glucoside	1.73883	6.242445
Naringenin	−4.92947	5.384106	Hesperetin*-O-*glucoside*-O-*HMG isomer 2	-0.6407	3.39011	Naringenin 7*-O-*rutinoside (Narirutin)	1.928781	6.68403
Bergaptol	−4.7894	3.991072	Hesperetin*-O-*glucoside isomer 2	-0.63071	3.43995	Chrysoeriol-7*-O-*neohesperidoside	1.92922	7.104414
Feruloyl glucoside isomer 1	−4.60201	4.521331	6-(beta-D-glucopyranosyloxy)-5-benzofuranpropanoic acid	-0.48622	2.878525	Hesperetin*-O-*glucoside*-O-*HMG isomer 1	1.948373	5.950696
Limonin	−4.53943	8.882311	Deacetylnomilin	-0.39546	3.456234	Luteolin*-O-*neohesperidoside*-O-*glucoside	2.01495	5.727777
Sinapoyl glucoside isomer 2	−4.36762	5.700092	Diosmetin-7*-O-*glucoside	-0.34885	2.732	Naringenin-C-glucoside-di*-O-*rhamnoside	2.245197	6.306151
Deacetylnomilinic acid	−4.22667	4.417491	Quinic acid	-0.32241	2.867993	Hesperetin 7*-O-*rutinoside (Hesperidin)	2.280418	7.023179
Luteolin-C-glucoside*-O-*rhamnoside	−4.14705	5.757555	Apigenin	-0.10009	0.9589588	Luteolin-7*-O-*glucoside	2.492401	7.271265
Isosakuranetin-7*-O-*neohesperidoside*-O-*HMG	−3.95891	2.074153	Brutieridin (Neohesperidin*-O-*HMG)	-0.05064	0.4115826	Unknown 2	2.503742	6.092729
6-(beta-D-glucopyranosyloxy)-4-methoxy-5-benzofuranpropanoic acid*-O-*HMG	−3.73521	4.878815	Bergamjuicin (Melitidin-glucoside)	-0.01964	0.1674981	Luteolin-6,8-di-C-glucoside	2.512266	5.605342
Nomilinic acid	−3.45355	4.106673	Diosmetin-8-C-glucoside	-0.01946	0.1436343	Eriodictyol-di*-O-*glucoside*-O-*HMG	2.536376	6.725588
Eriodictyol	−2.41865	5.103139	Naringenin 7*-O-*neohesperidoside (Naringin)	0.014335	0.09862396	Diosmetin-acetyl*-O-*neohesperidoside	2.749035	4.671219
Apigenin-di*-O-*glucoside*-O-*HMG	−2.33467	5.142885	255-C-glucoside*-O-*rhamnoside	0.047444	0.5890086	Neoeriocitrin*-O-*glucoside/eriocitrin*-O-*glucoside	3.098905	6.850492
6-(beta-D-glucopyranosyloxy)-4-methoxy-5-benzofuranpropanoic acid	−2.25969	4.728437	Apigenin-6,8-di-C-glucoside	0.058434	0.5895315	Apigenin*-O-*glucoside	3.182595	8.802576
Diosmetin-di*-O-*glucoside*-O-*HMG	−1.6841	4.828777	Luteolin-7*-O-*neohesperidoside*-O-*HMG	0.063004	0.5041117	Luteolin*-O-*acetyl*-O-*neohesperidoside	3.400251	7.082
Chrysoeriol-di*-O-*glucoside*-O-*HMG	−1.38935	2.543526	Naringin-glucoside	0.065004	0.5444015	Luteolin	3.805598	5.703613
Naringenin-7*-O-*glucoside (Prunasin)	−1.30443	4.462694	Apigenin-7*-O-*neohesperidoside	0.140053	1.148525	Unknown 3	3.963885	7.479016
Eriocitrin*-O-*HMG	−1.26234	3.851215	Diosmetin-6,8-di-C-glucoside	0.214394	2.027318	Luteolin-7*-O-*rutinoside	4.174565	6.688246
2-Hydroxy-4-methoxyhydrocinnamoyl-2*-O-*glucoside	−1.23	4.366283	Hesperetin 7*-O-*neohesperidoside (Neohesperidin)	0.225147	2.50244	Hesperetin*-O-*rutinoside*-O-*HMG	5.059471	7.079574
Demethoxycentaureidin-7*-O-*glucoside	−1.16319	4.184481	Eriodictyol 7*-O-*neohesperidoside (Neoeriocitrin)	0.230102	2.337394	Luteolin*-O-*neohesperidoside*-O-*rhamnoside	5.288257	7.189358
Eriodictyol*-O-*glucoside*-O-*HMG	−1.15424	4.374825	Eriodictyol-7*-O-*glucoside	0.420067	3.020995			
Naringenin*-O-*rutinoside*-O-*HMG	−1.14268	4.42741	Hesperetin-di*-O-*glucoside*-O-*HMG	0.454134	3.281			
Demethoxycentaureidin-7*-O-*glucoside*-O-*HMG	−1.09035	4.207735	Melitidin (Naringin*-O-*HMG)	0.501746	3.635704			
			3-[2,4,5-trihydroxy-3-(3-methylbut-2-en-1-yl)phenyl]propanoic acid	0.540642	4.369824			
			Chrysoeriol*-O-*glucoside*-O-*HMG	0.592265	3.118346			
			Neoeriocitrin*-O-*HMG	0.618654	3.886167			
			Unknown 1	0.696112	4.537258			
			255-neohesperidoside*-O-*HMG	0.705438	4.290545			
			315-glucoside*-O-*HMG (2)	0.779496	4.644788			
			Apigenin-6-C-glucoside	0.817564	3.956206			
			Diosmetin-7*-O-*neohesperidoside	0.900638	4.928689			
			Eriodictyol 7*-O-*rutinoside (Eriocitrin)	0.931918	4.711979			
			Chrysoeriol-6,8-di-C-glucoside	0.989296	4.380177			

**Table 5:** Content (mg/g extract) of the major flavonoids present in the extracts expressed as mean (standard deviation).

Extract	Neoeriocitrin	Naringin	Neohesperidin	Melitidin	Brutieridin
B**F**PF	94.72 (0.20)	38.05 (0.09)	20.32 (0.02)	22.84 (0.11)	30.16 (0.52)
B**L**PF	133.42 (3.22)	164.31 (1.48)	144.95 (1.36)	31.2 (2.19)	59.77 (2.04)

**Table 6:** Total phenolic content and antioxidant activity mean values (± SD) of the two tested extracts, absolute values and values normalized on total phenolic content.

Extract	Folin-Ciocalteu mg Gallic acid/g Extract	ORAC µmol Trolox/g Extract	DPPH IC_50_ (µg/mL)	ABTS IC_50_ (µg/mL)
BFPF	167.3 ± 12.5	9834 ± 542	42.2 ± 1.7	96.3 ± 7.3
BLPF	230.3 ± 31.7	13145 ± 3487	20.1 ± 1.3	49.5 ± 5.6
Ascorbic acid		4318	41.2	28.2
**Extract Normalized Values**		**Orac** **µmol Trolox/Mg Gallic Acid**	**DPPH** **IC_50_ (µg Gallic Acid/mL)**	**ABTS** **IC_50_ (µg Gallic Acid/mL)**
BFPF		63.6 ± 11.2	7.07 ± 0.74	16.2 ± 2.4
BLPF		59.3 ± 25.1	4.65 ± 0.9	11.5 ± 2.8

## Data Availability

The data presented in this study are available on request from the corresponding author. The data are not publicly available.

## Supplementary Materials

The following are available online at https://www.mdpi.com/2076-3921/10/2/141/s1, Method S1: Method validation, Table S1: Database for the targeted analysis, Figure S1: Quinic acid, Figure S2: HMG*-O-*glucoside, Figure S3: 6-(beta-D-glucopyranosyloxy)-5-benzofuranpropanoic acid, Figure S4: 6-(beta-D-glucopyranosyloxy)-4-methoxy-5-benzofuranpropanoic acid, Figure S5: 6-hydroxy-4-methoxy-5-benzofuranpropanoic acid, Figure S6: Bergaptol, Figure S7: Deacetylnomilinic acid, Figure S8: Limonoate A-ring lactone, Figure S9: Deacetylnomilin, Figure S10: p-coumaric acid, Figure S11: 3-[2,4,5-trihydroxy-3-(3-methylbut-2-en-1-yl)phenyl]propanoic acid, Figure S12: Neoeriocitrin*-O-*glucoside/eriocitrin*-O-*glucoside, Figure S13: Luteolin*-O-*neohesperidoside*-O-*glucoside, Figure S14: Luteolin-C-glucoside*-O-*rhamnoside, Figure S15: Luteolin*-O-*rutinoside, Figure S16: Luteolin*-O-*neohesperidoside*-O-*rhamnoside, Figure S17: Hesperetin-di-C-glucoside, Figure S18: Naringenin-C-glucoside-di*-O-*rhamnoside, Figure S19: Apigenin*-O-*glucoside, Figure S20: Luteolin*-O-*acetyl*-O-*neohesperidoside, Figure S21: Naringenin-C-glucoside-di*-O-*rhamnoside, Figure S22: Diosmetin*-O-*acetyl-neohesperidoside, Figure S23: Unknown 1, Figure S24: 255-C-glucoside*-O-*rhamnoside, Figure S25: Unknown 2, Figure S26: Unknown 3, Figure S27: UV trace of the LeucoselectTM GPC, Figure S28: UV trace of the BFPF GPC, Figure S29. UV trace of the BLPF GPC, Figure S30: Evaluation of tannin effect.

## References

[1] Cautela D., Vella F.M., Laratta B. (2019). The Effect of Processing Methods on Phytochemical Composition in Bergamot Juice. Foods.

[2] Mannucci C., Navarra M., Calapai F., Squeri R., Gangemi S., Calapai G. (2017). Clinical Pharmacology of Citrus bergamia: A Systematic Review. Phytother. Res..

[3] Giuffrè A.M. (2019). Bergamot (Citrus bergamia, Risso): The Effects of Cultivar and Harvest Date on Functional Properties of Juice and Cloudy Juice. Antioxidants.

[4] Carresi C., Gliozzi M., Musolino V., Scicchitano M., Scarano F., Bosco F., Nucera S., Maiuolo J., Macrì R., Ruga S. (2020). The Effect of Natural Antioxidants in the Development of Metabolic Syndrome: Focus on Bergamot Polyphenolic Fraction. Nutrients.

[5] Lamiquiz-Moneo I., Giné-González J., Alisente S., Bea A.M., Pérez-Calahorra S., Marco-Benedí V., Baila-Rueda L., Jarauta E., Cenarro A., Civeira F. (2020). Effect of bergamot on lipid profile in humans: A systematic review. Crit. Rev. Food Sci. Nutr..

[6] Nauman M.C., Johnson J.J. (2019). Clinical application of bergamot (Citrus bergamia) for reducing high cholesterol and cardiovascular disease markers. Integr. Food Nutr. Metab..

[7] Mollace V., Sacco I., Janda E., Malara C., Ventrice D., Colica C., Visalli V., Muscoli S., Ragusa S., Muscoli C. (2011). Hypolipemic and hypoglycaemic activity of bergamot polyphenols: From animal models to human studies. Fitoterapia.

[8] Musolino V., Gliozzi M., Scarano F., Bosco F., Scicchitano M., Nucera S., Carresi C., Ruga S., Zito M.C., Maiuolo J. (2020). Bergamot Polyphenols Improve Dyslipidemia and Pathophysiological Features in a Mouse Model of Non-Alcoholic Fatty Liver Disease. Sci. Rep..

[9] De Leo M., Piragine E., Pirone A., Braca A., Pistelli L., Calderone V., Miragliotta V., Testai L. (2020). Protective Effects of Bergamot (Citrus bergamia Risso & Poiteau) Juice in Rats Fed with High-Fat Diet. Planta Med..

[10] Mollace V., Scicchitano M., Paone S., Casale F., Calandruccio C., Gliozzi M., Musolino V., Carresi C., Maiuolo J., Nucera S. (2019). Hypoglycemic and Hypolipemic Effects of a New Lecithin Formulation of Bergamot Polyphenolic Fraction: A Double Blind, Randomized, Placebo Controlled Study. Endocr. Metab. Immune Disord. Drug Targets.

[11] Leopoldini M., Malaj N., Toscano M., Sindona G., Russo N. (2010). On the inhibitor effects of bergamot juice flavonoids binding to the 3-hydroxy-3-methylglutaryl-CoA reductase (HMGR) enzyme. J. Agric. Food Chem..

[12] Ballistreri G., Amenta M., Fabroni S., Consoli V., Grosso S., Vanella L., Sorrenti V., Rapisarda P. (2020). Evaluation of lipid and cholesterol-lowering effect of bioflavonoids from bergamot extract. Nat. Prod. Res..

[13] Ferlemi A.V., Lamari F.N. (2016). Berry Leaves: An Alternative Source of Bioactive Natural Products of Nutritional and Medicinal Value. Antioxidants.

[14] Baron G., Altomare A., Regazzoni L., Fumagalli L., Artasensi A., Borghi E., Ottaviano E., Del Bo C., Riso P., Allegrini P. (2020). Profiling Vaccinium macrocarpon components and metabolites in human urine and the urine ex-vivo effect on Candida albicans adhesion and biofilm-formation. Biochem. Pharmacol..

[15] Risitano R., Currò M., Cirmi S., Ferlazzo N., Campiglia P., Caccamo D., Ientile R., Navarra M. (2014). Flavonoid fraction of Bergamot juice reduces LPS-induced inflammatory response through SIRT1-mediated NF-κB inhibition in THP-1 monocytes. PLoS ONE.

[16] Janda E., Salerno R., Martino C., Lascala A., La Russa D., Oliverio M. (2018). Qualitative and quantitative analysis of the proautophagic activity of Citrus flavonoids from Bergamot Polyphenol Fraction. Data Brief.

[17] Mandalari G., Bennett R.N., Bisignano G., Saija A., Dugo G., Lo Curto R.B., Faulds C.B., Waldron K.W. (2006). Characterization of flavonoids and pectins from bergamot (Citrus bergamia Risso) peel, a major byproduct of essential oil extraction. J. Agric. Food Chem..

[18] Gattuso G., Barreca D., Caristi C., Gargiulli C., Leuzzi U. (2007). Distribution of flavonoids and furocoumarins in juices from cultivars of Citrus bergamia Risso. J. Agric. Food Chem..

[19] Gattuso G., Caristi C., Gargiulli C., Bellocco E., Toscano G., Leuzzi U. (2006). Flavonoid glycosides in bergamot juice (Citrus bergamia Risso). J. Agric. Food Chem..

[20] Da Pozzo E., De Leo M., Faraone I., Milella L., Cavallini C., Piragine E., Testai L., Calderone V., Pistelli L., Braca A. (2018). Antioxidant and Antisenescence Effects of Bergamot Juice. Oxidative Med. Cell. Longev..

[21] Gardana C., Nalin F., Simonetti P. (2008). Evaluation of flavonoids and furanocoumarins from Citrus bergamia (Bergamot) juice and identification of new compounds. Molecules.

[22] Spigoni V., Mena P., Fantuzzi F., Tassotti M., Brighenti F., Bonadonna R.C., Del Rio D., Dei Cas A. (2017). Bioavailability of Bergamot (*Citrus bergamia*) Flavanones and Biological Activity of Their Circulating Metabolites in Human Pro-Angiogenic Cells. Nutrients.

[23] Russo M., Arigò A., Calabrò M.L., Farnetti S., Mondello L., Dugo P. (2016). Bergamot (Citrus bergamia Risso) as a source of nutraceuticals: Limonoids and flavonoids. J. Funct. Foods.

[24] Salerno R., Casale F., Calandruccio C., Procopio A. (2016). Characterization of flavonoids in Citrus bergamia (Bergamot) polyphenolic fraction by liquid chromatography–high resolution mass spectrometry (LC/HRMS). PharmaNutrition.

[25] Formisano C., Rigano D., Lopatriello A., Sirignano C., Ramaschi G., Arnoldi L., Riva A., Sardone N., Taglialatela-Scafati O. (2019). Detailed Phytochemical Characterization of Bergamot Polyphenolic Fraction (BPF) by UPLC-DAD-MS and LC-NMR. J. Agric. Food Chem..

[26] Gabetta B., Fuzzati N., Griffini A., Lolla E., Pace R., Ruffilli T., Peterlongo F. (2000). Characterization of proanthocyanidins from grape seeds. Fitoterapia.

[27] Baron G., Altomare A., Fumagalli L., Rumio C., Carini M., Vistoli G., Aldini G. (2019). Development of a direct ESI-MS method for measuring the tannin precipitation effect of proline-rich peptides and in silico studies on the proline role in tannin-protein interactions. Fitoterapia.

[28] Dewanto V., Wu X., Adom K.K., Liu R.H. (2002). Thermal processing enhances the nutritional value of tomatoes by increasing total antioxidant activity. J. Agric. Food Chem..

[29] Wang H., Cao G., Prior R.L. (1997). Oxygen Radical Absorbing Capacity of Anthocyanins. J. Agric. Food Chem..

[30] Re R., Pellegrini N., Proteggente A., Pannala A., Yang M., Rice-Evans C. (1999). Antioxidant activity applying an improved ABTS radical cation decolorization assay. Free Radic. Biol. Med..

[31] Brand-Williams W., Cuvelier M.E., Berset C. (1995). Use of a free radical method to evaluate antioxidant activity. LWT-Food Sci. Technol..

[32] Scavello F., Zeni F., Tedesco C.C., Mensà E., Veglia F., Procopio A.D., Bonfigli A.R., Olivieri F., Raucci A. (2019). Modulation of soluble receptor for advanced glycation end-products (RAGE) isoforms and their ligands in healthy aging. Aging.

[33] Zeuner M.T., Vallance T., Vaiyapuri S., Cottrell G.S., Widera D. (2017). Development and Characterisation of a Novel NF-κB reporter cell line for investigation of neuroinflammation. Mediat. Inflamm..

[34] Allen F., Pon A., Wilson M., Greiner R., Wishart D. (2014). CFM-ID: A web server for annotation, spectrum prediction and metabolite identification from tandem mass spectra. Nucleic Acids Res..

[35] Aldini G., Regazzoni L., Pedretti A., Carini M., Cho S.M., Park K.M., Yeum K.J. (2011). An integrated high resolution mass spectrometric and informatics approach for the rapid identification of phenolics in plant extract. J. Chromatogr. A.

[36] Chen C.C., Huang Y.L., Huang F.I., Wang C.W., Ou J.C. (2001). Water-soluble glycosides from Ruta graveolens. J. Nat. Prod..

[37] Pacifico S., Piccolella S., Galasso S., Fiorentino A., Kretschmer N., Pan S.P., Bauer R., Monaco P. (2016). Influence of harvest season on chemical composition and bioactivity of wild rue plant hydroalcoholic extracts. Food Chem. Toxicol..

[38] Abad-García B., Garmón-Lobato S., Berrueta L.A., Gallo B., Vicente F. (2012). On line characterization of 58 phenolic compounds in Citrus fruit juices from Spanish cultivars by high-performance liquid chromatography with photodiode-array detection coupled to electrospray ionization triple quadrupole mass spectrometry. Talanta.

[39] He Y., Li Z., Wang W., Sooranna S.R., Shi Y., Chen Y., Wu C., Zeng J., Tang Q., Xie H. (2018). Chemical Profiles and Simultaneous Quantification of *Aurantii fructus* by Use of HPLC-Q-TOF-MS Combined with GC-MS and HPLC Methods. Molecules.

[40] Fujii Y., Tanaka R., Miyake H., Tamaru Y., Ueda M., Shibata T. (2013). Evaluation for Antioxidative Properties of Phlorotannins Isolated from the Brown Alga Eisenia bicyclis, by the H-ORAC Method. Food Nutr. Sci..

[41] Mishra K., Ojha H., Chaudhury N.K. (2012). Estimation of antiradical properties of antioxidants using DPPH assay: A critical review and results. Food Chem..

[42] Matuszewska A., Jaszek M., Stefaniuk D., Ciszewski T., Matuszewski Ł. (2018). Anticancer, antioxidant, and antibacterial activities of low molecular weight bioactive subfractions isolated from cultures of wood degrading fungus Cerrena unicolor. PLoS ONE.

