# Supplementary material for: Analytical Profile and Antioxidant and Anti-Inflammatory Activities of the Enriched Polyphenol Fractions Isolated from Bergamot Fruit and Leave
Source: Antioxidants (Basel). 2021 Jan 20;10(2):141. doi: 10.3390/antiox10020141 (PMC7908980; doi:10.3390/antiox10020141)
Supplement: Supplementary file 1 [file antioxidants-10-00141-s001.pdf]

Table S1. Database for the targeted analysis.

Name	Other name	Molecular Formula	Exact monoisotopic mass	References
2-O-β-glucopyranose-2-hydroxy-4-methoxyhydrocinnamic acid	2-Hydroxy-4-methoxyhydrocinnamoyl-2-O-glucoside	$C_{16}H_{22}O_9$	358.1264	[25]
5-geranoxy-7-methoxycoumarin		$C_{20}H_{24}O_4$	328.1675	[17,23]
5-Sinapoylquinic acid		$C_{18}H_{22}O_{10}$	398.1213	[9,23]
Apigenin 6,8 di C-glucoside	Vicenin-2	$C_{27}H_{30}O_{15}$	594.1585	[9,15,17-21,23-25]
Apigenin 6-C-glucoside		$C_{21}H_{20}O_{10}$	432.1056	[18-19,21,25]
Apigenin 7-O- diglucuronide		$C_{27}H_{26}O_{17}$	622.1170	[23]
Apigenin 7-O-neohesperidoside	Rhoifolin	$C_{27}H_{30}O_{14}$	578.1636	[15-16,18-19,21,23-25]
Apigenin 7-O-neohesperidoside-4′-glucoside	Rhoifolin 4'-glucoside	$C_{33}H_{40}O_{19}$	740.2164	[15,18-19,23]
Apigenin monorhamnoside		$C_{21}H_{20}O_9$	416.1107	[17]
Apigenin-7-O-neohesperidoside-6''-O-HMG		$C_{33}H_{38}O_{18}$	722.2058	[16,24]
Apigenin-8-C-glucoside		$C_{21}H_{20}O_{10}$	432.1056	[9,21,25]
Apigenin		$C_{15}H_{10}O_5$	270.0528	[16]
Bergamjuicin		$C_{39}H_{50}O_{23}$	886.2743	[9,25]
Bergamottin		$C_{21}H_{22}O_4$	338.1518	[17-18,21,23,25]
Bergapten		$C_{12}H_8O_4$	216.0423	[9,17-18,21,23,25]
Brutieridin		$C_{34}H_{42}O_{19}$	754.2320	[9,15-16,20-25]
Chrysoeriol		$C_{16}H_{12}O_6$	300.0634	[16]
Chrysoeriol 6,8-di-C-glucoside	Stellarin-2	$C_{28}H_{32}O_{16}$	624.1690	[18-19,21,25]
Chrysoeriol 7-O-neohesperidoside		$C_{28}H_{32}O_{15}$	608.1741	[9,18-21]
Chrysoeriol 7-O-neohesperidoside- 4′-glucoside		$C_{34}H_{42}O_{20}$	770.2269	[18-19]

Chrysoeriol 8-C-glucoside	Scoparin	$C_{22}H_{22}O_{11}$	462.1162	[18-19,21,24-25]
Chrysoeriol-O-glucoside/Diosmetin-O-glucoside		$C_{22}H_{22}O_{11}$	462.1162	[25]
Citric acid		$C_6H_8O_7$	192.0270	[9,20]
Citropten		$C_{11}H_{10}O_4$	206.0579	[23]
Diosmetin-6,8-di-C-glucoside	Lucenin-2 4'-methyl ether	$C_{28}H_{32}O_{16}$	624.1690	[9,15,17-21,23-25]
Deacetyl nomilin glucoside		$C_{32}H_{44}O_{14}$	652.2731	[23]
Deacetyl nomilinic acid glucoside		$C_{32}H_{46}O_{15}$	670.2837	[23]
Demethoxycentaureidin-7-O-β-glucoside		$C_{23}H_{24}O_{12}$	492.1268	[25]
Demethoxycentaureidin-7-O-β-glucoside-HMG		$C_{29}H_{32}O_{16}$	636.1690	[25]
Di-acetyl Caffeic acid		$C_{13}H_{12}O_6$	264.0634	[21]
Diosmetin 7-O-neohesperidoside	Neodiosmin	$C_{28}H_{32}O_{15}$	608.1741	[9,15-16,18-21,23-25]
Diosmetin 8-C-glucoside	Orientin 4' methylether	$C_{22}H_{22}O_{11}$	462.1162	[9,15,17-19,23-25]
Diosmetin mono-rhamnoside		$C_{22}H_{22}O_{10}$	446.1213	[17]
Diosmetin-7-O-glucoside		$C_{22}H_{22}O_{11}$	462.1162	[16-17,24]
Diosmetin-7-O-neohesperidoside-6''-O-HMG		$C_{34}H_{40}O_{19}$	752.2164	[16,24]
Diosmetin-7-O-rutinoside	Diosmin	$C_{28}H_{32}O_{15}$	608.1741	[16]
Diosmetin		$C_{16}H_{12}O_6$	300.0634	[15]
Eriodictyol 7-O-rutinoside	Eriocitrin	$C_{27}H_{32}O_{15}$	596.1741	[9,15,17,19,21-23,25]
Eriodictyol 7-O-neohesperidoside	Neoeriocitrin	$C_{27}H_{32}O_{15}$	596.1741	[9,15-25]
Eriodictyol mono-rhamnoside		$C_{21}H_{22}O_{10}$	434.1213	[17]
Eriodictyol-7-O-neohesperidoside-6″-O-HMG	Neoeriocitrin-O-HMG	$C_{33}H_{40}O_{19}$	740.2164	[9,16,20-22,24]
Eriodictyol-7-O-neohesperidoside-6″-O-HMG-glucoside		$C_{39}H_{50}O_{24}$	902.2692	[9]

eriodictyol-7-O-β-glucoside		$C_{21}H_{22}O_{11}$	450.1162	[25]
Eriodyctiol		$C_{15}H_{12}O_6$	288.0634	[22,25]
Ferulic acid 4-O-glucoside		$C_{16}H_{20}O_9$	356.1107	[21,23]
Heptamethoxyflavone		$C_{22}H_{24}O_9$	432.1420	[23]
Hesperetin		$C_{16}H_{14}O_6$	302.0790	[15,22,25]
Hesperetin-7-O-neohesperidoside	Neohesperidin	$C_{28}H_{34}O_{15}$	610.1898	[9,15-25]
hesperetin mono-rhamnoside		$C_{22}H_{24}O_{10}$	448.1369	[17]
Hesperetin-7-O-glucoside-6''-O-HMG		$C_{28}H_{32}O_{15}$	608.1741	[16,24]
Hesperetin-7-O-rutinoside	Hesperidin	$C_{28}H_{34}O_{15}$	610.1898	[15,22]
Hesperetin-7-O-β-glucoside		$C_{22}H_{24}O_{11}$	464.1319	[16,24-25]
Hesperetin-7-O-neohesperidoside-6''-O-HMG-glucoside	Neohesperidin-O-HMG glucoside	$C_{40}H_{52}O_{24}$	916.2848	[9]
Ichangin		$C_{26}H_{32}O_9$	488.2046	[23]
Isosakuranetin		$C_{16}H_{14}O_5$	286.0841	[22]
Isosakuranetin-7-O-neohesperidoside-6″-O-HMG	Parmigin	$C_{34}H_{42}O_{18}$	738.2371	[22]
Isosakuranetin-7-O-rutinoside	Didymin	$C_{28}H_{34}O_{14}$	594.1949	[22]
Isosakuranetin-7-neohesperidoside	Poncirin	$C_{28}H_{34}O_{14}$	594.1949	[15,23]
Kaempferol 3-O-xylosyl-glucoside		$C_{26}H_{28}O_{15}$	580.1428	[23]
Limonin		$C_{26}H_{30}O_8$	470.1941	[9,23,25]
Limonin glucoside		$C_{32}H_{42}O_{14}$	650.2574	[20,23]
Luteolin		$C_{15}H_{10}O_6$	286.0477	[16]
Luteolin-8-C-glucoside	Orientin	$C_{21}H_{20}O_{11}$	448.1006	[21]
Luteolin 6,8-di-C-glucoside	Lucenin-2	$C_{27}H_{30}O_{16}$	610.1534	[18-19,21,24-25]

Luteolin-7-O-neohesperidoside		$C_{27}H_{30}O_{15}$	594.1585	[9,16,24-25]
Luteolin mono-glucoside/monorhamnoside		$C_{21}H_{20}O_{11}$/$C_{21}H_{20}O_{10}$	448.10056/432.10564	[17]
Naringenin 7-O-neohesperidoside-6''-O-HMG	Melitidin	$C_{33}H_{40}O_{18}$	724.2215	[9,15-16,20-25]
Naringenin		$C_{15}H_{12}O_5$	272.0685	[15,22,25]
Naringenin 7-O-neohesperidoside	Naringin	$C_{27}H_{32}O_{14}$	580.1792	[9,15-25]
Naringenin mono-rhamnoside		$C_{21}H_{22}O_9$	418.1264	[17]
Naringenin-7-O-glucoside-6″-O-HMG		$C_{27}H_{30}O_{14}$	578.1636	[16,22,24]
Naringenin-7-O-rutinoside	Narirutin	$C_{27}H_{32}O_{14}$	578.1636	[15,17,22-23,25]
Naringenin-7-O-β-glucopyranoside	Prunasin	$C_{21}H_{22}O_{10}$	434.1213	[25]
Naringin-4′-O-β-glucopyranoside		$C_{33}H_{42}O_{19}$	742.2320	[25]
Nobiletin		$C_{21}H_{22}O_8$	402.1315	[23]
Nomilin		$C_{28}H_{34}O_9$	514.2203	[23]
Nomilin glucoside		$C_{34}H_{46}O_{15}$	694.2837	[23]
Nomilinic acid		$C_{28}H_{36}O_{10}$	532.2308	[9,25]
Nomilinic acid glucoside		$C_{34}H_{48}O_{16}$	712.2942	[9,20,23,25]
Obacunone glucoside		$C_{32}H_{42}O_{13}$	634.2625	[23]
Quercetin-3-b-glucopyranoside	Isoquercitrin	$C_{21}H_{20}O_{12}$	464.0955	[15]
Quercetin rutinoside	Rutin	$C_{27}H_{30}O_{16}$	610.1534	[25]
Sinapoyl glucose		$C_{17}H_{22}O_{10}$	386.1213	[21,23]
Sinensetin		$C_{20}H_{20}O_7$	372.1209	[23,25]
Tangeretin		$C_{20}H_{20}O_7$	372.1209	[23,25]
Tetra-O-methylscutellarein		$C_{19}H_{18}O_6$	342.1103	[23]

Umbelliferone glucoside	$C_{15}H_{16}O_8$	324.0845	[25]
Verbascoside	$C_{29}H_{36}O_{15}$	624.2054	[23]
α-Limonol or Obacunoic acid	$C_{26}H_{32}O_8$	472.2097	[23]

S1. Method validation

The major flavonoids (neoeriocitrin, naringin, neohesperidin, melitidin, and brutieridin) present in bergamot extracts were determined by chromatographic analysis with an HPLC system equipped with a DAD detector. Since the HPLC method was calibrated using naringin as standard, their concentration is expressed as naringin equivalent (mg/g extract). The calibration curve of naringenin was built in the range 12-300 mg/g extract by plotting the peak area of naringin versus the nominal concentrations by weighted ($1/x^2$) least-squares linear regression: $y=1574(\pm 25.65)x - 10551(\pm 3898)$, $r^2=0.9989$. The concentration 12 mg/g was considered as the LLOQ because the precision (CV%) and accuracy (bias %) were lower than ±20%: +2.9 % and +17.1%, for CV% and bias % respectively. The precision and accuracy were calculated on five replicates of 3 concentrations: for the LLOQ the CV% and bias % were reported above, the precision and accuracy for the concentration 80 mg/g were +4.7% and -8.6%, and for the concentration 300 mg/g they were +2.2% and -0.4%, respectively. The method was considered selective because no peak of analyte was present at the same retention time of the analytes in 6 different blank samples.

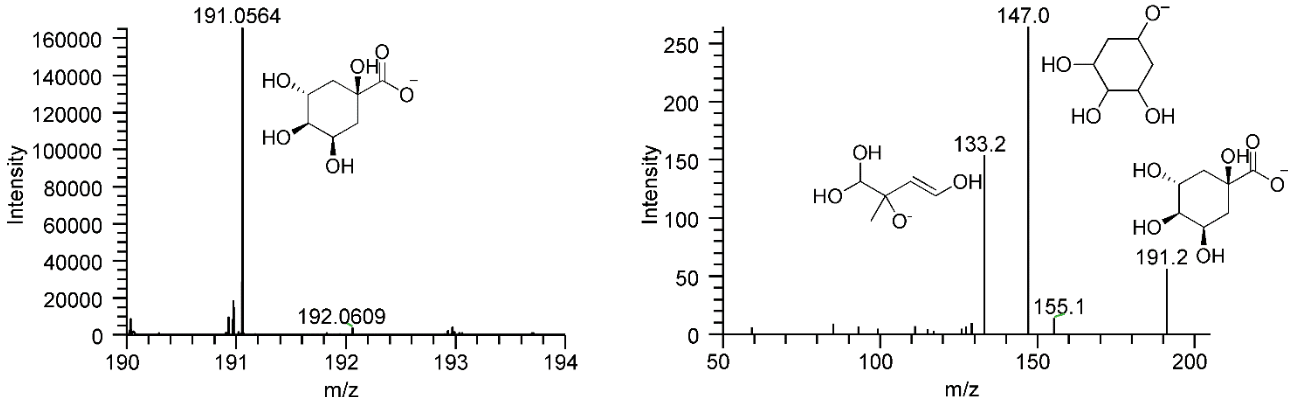

Figure S1. **Quinic acid**: $C_7H_{12}O_6$ *m/z* 191.0564. MS/MS: *m/z* 147, 133. Found in MoNa and HMDB databases. Fragmentation confirmed by the Peak assignment tool of CFM-ID.

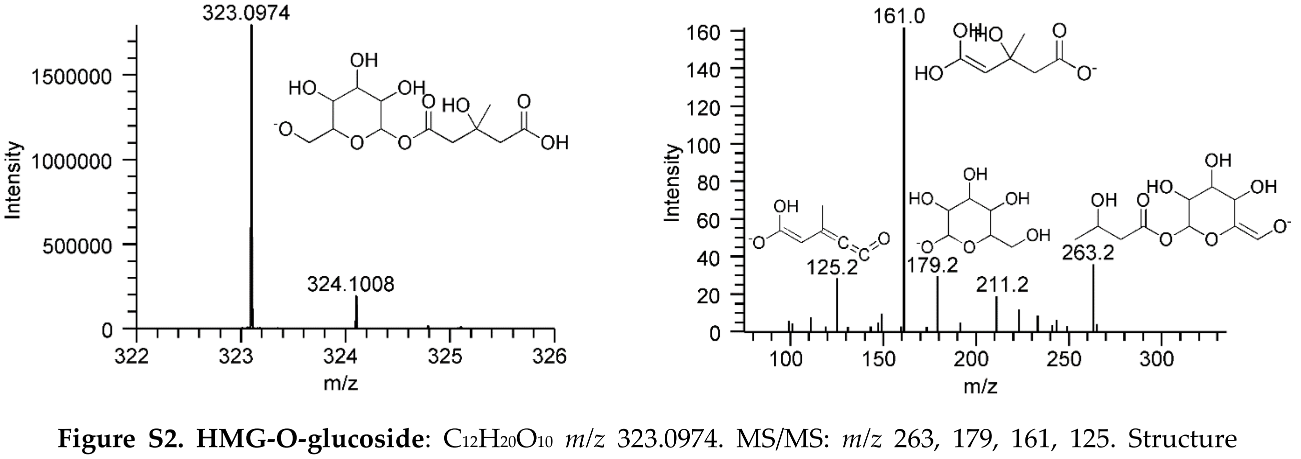

Figure S2. HMG-O-glucoside: $C_{12}H_{20}O_{10}$ *m/z* 323.0974. MS/MS: *m/z* 263, 179, 161, 125. Structure hypothesized through the mass losses and confirmed by the Peak assignment tool of CFM-ID

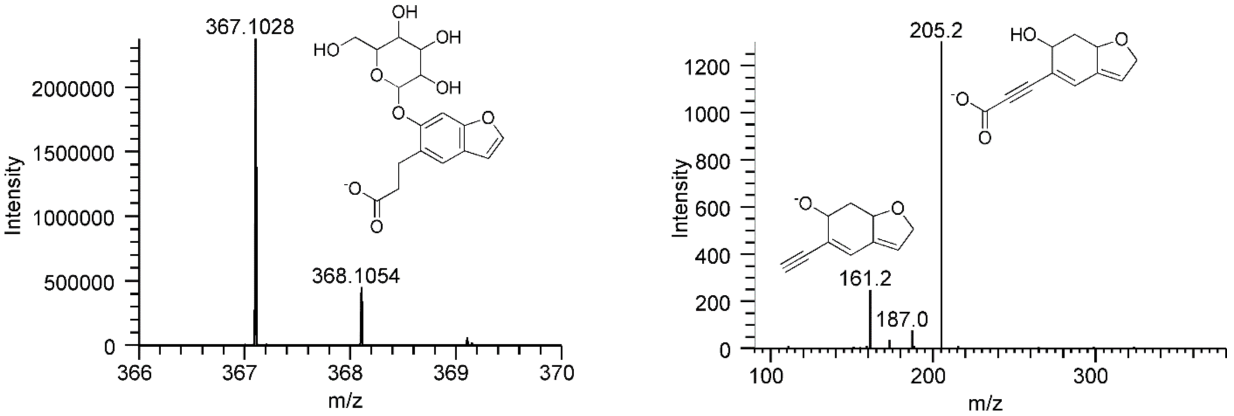

Figure S3. **6-(beta-D-glucopyranosyloxy)-5-benzofuranpropanoic acid**: $C_{17}H_{20}O_9$ *m/z* 367.1028. MS/MS: *m/z* 205, 161. Found through the Spectra Similarity Search of MoNa database.

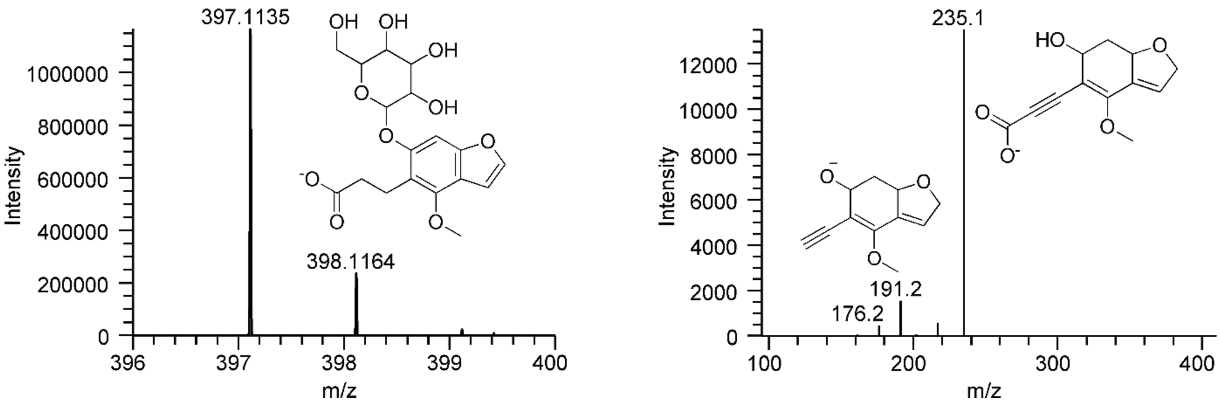

Figure S4. **6-(beta-D-glucopyranosyloxy)-4-methoxy-5-benzofuranpropanoic acid**: $C_{18}H_{22}O_{10}$ *m/z* 397.1135. MS/MS: *m/z* 235, 191. Found in MoNa database. Fragmentation confirmed by the Peak assignment tool of CFM-ID.

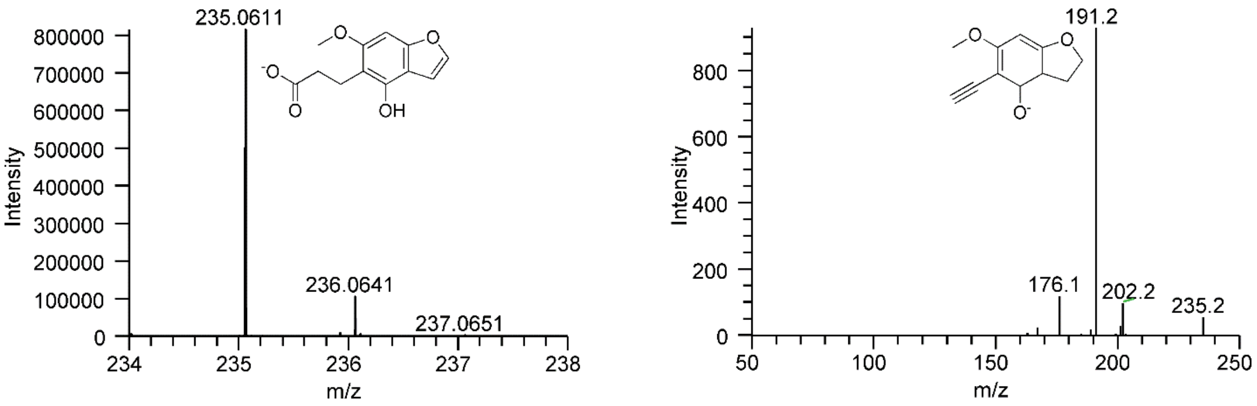

Figure S5. **6-hydroxy-4-methoxy-5-benzofuranpropanoic acid**: $C_{12}H_{12}O_5$ *m/z* 235.0611. MS/MS: *m/z* 191, 176. Hypothesized to be the aglycone of 6-(beta-D-glucopyranosyloxy)-4-methoxy-5-benzofuranpropanoic acid. Fragmentation confirmed by the Peak assignment tool of CFM-ID.

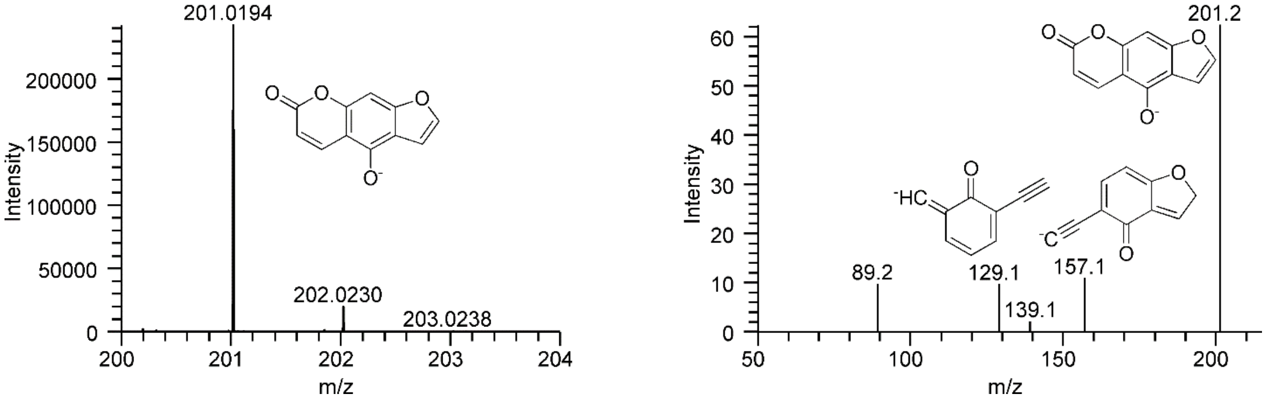

Figure S6. **Bergaptol**: $C_{11}H_6O_4$ *m/z* 201.0194. MS/MS: *m/z* 157. Found in HMDB database. Fragmentation confirmed by the Peak assignment tool of CFM-ID.

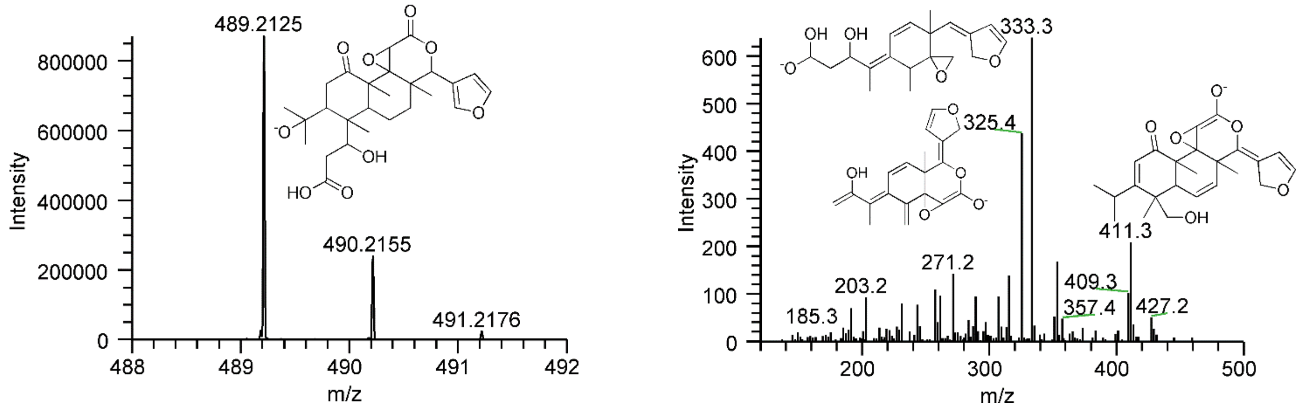

Figure S7. **Deacetylnomilinic acid**: $C_{26}H_{34}O_9$ *m/z* 489.2125. MS/MS: *m/z* 411, 333, 325. Found in HMDB database. Fragmentation confirmed by the Peak assignment tool of CFM-ID.

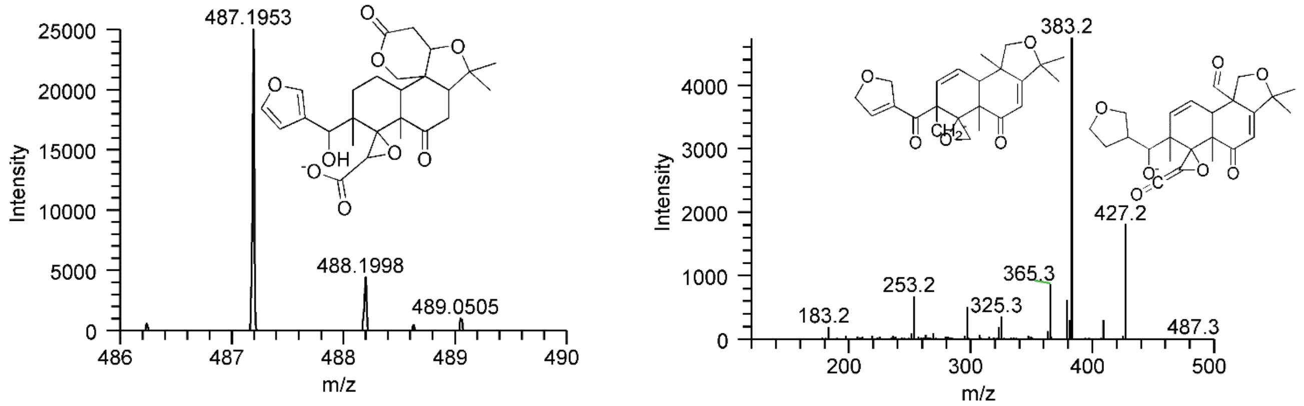

Figure S8. **Limonoate A-ring lactone**: $C_{26}H_{32}O_9$ *m/z* 487.1953. MS/MS: *m/z* 427, 383. Found in PubChem database. Fragmentation confirmed by the Peak assignment tool of CFM-ID.

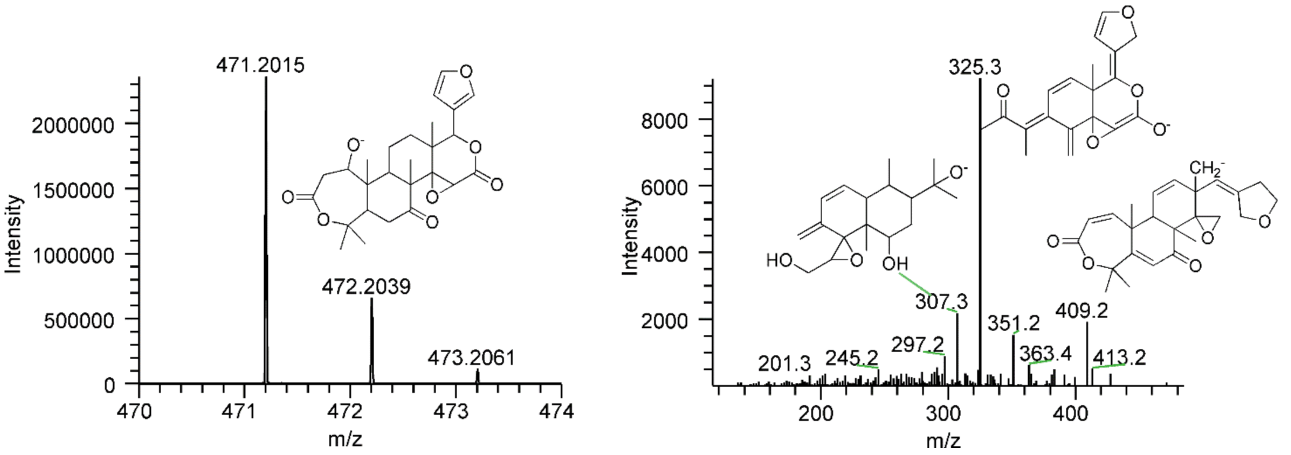

Figure S9. **Deacetylnomilin**: $C_{26}H_{32}O_8$ *m/z* 471.2015. MS/MS: *m/z* 409, 325, 307. Found in HMDB database. Fragmentation confirmed by the Peak assignment tool of CFM-ID.

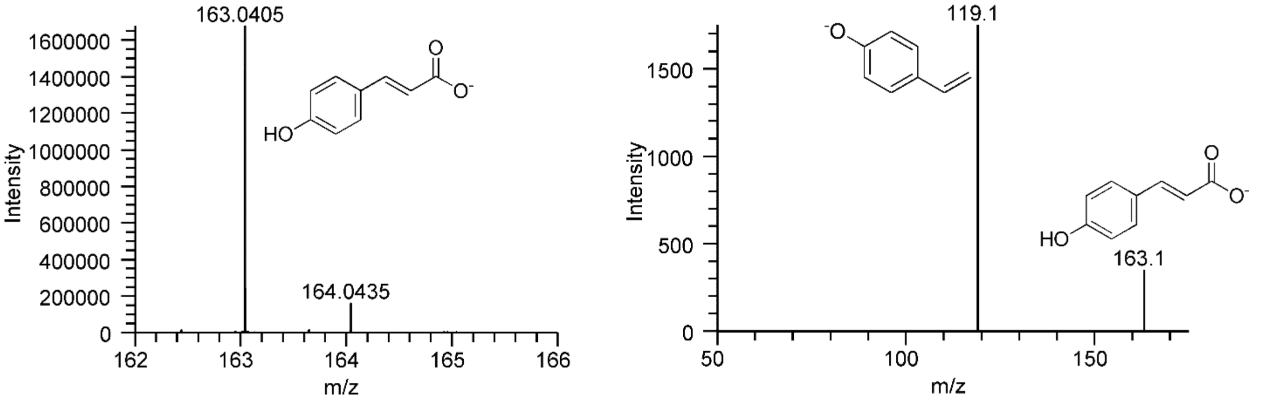

Figure S10. ***p*-coumaric acid**: $C_9H_8O_3$ m/z 163.0406. MS/MS: m/z 119. Found in MoNa and HMDB databases. Fragmentation confirmed by the Peak assignment tool of CFM-ID.

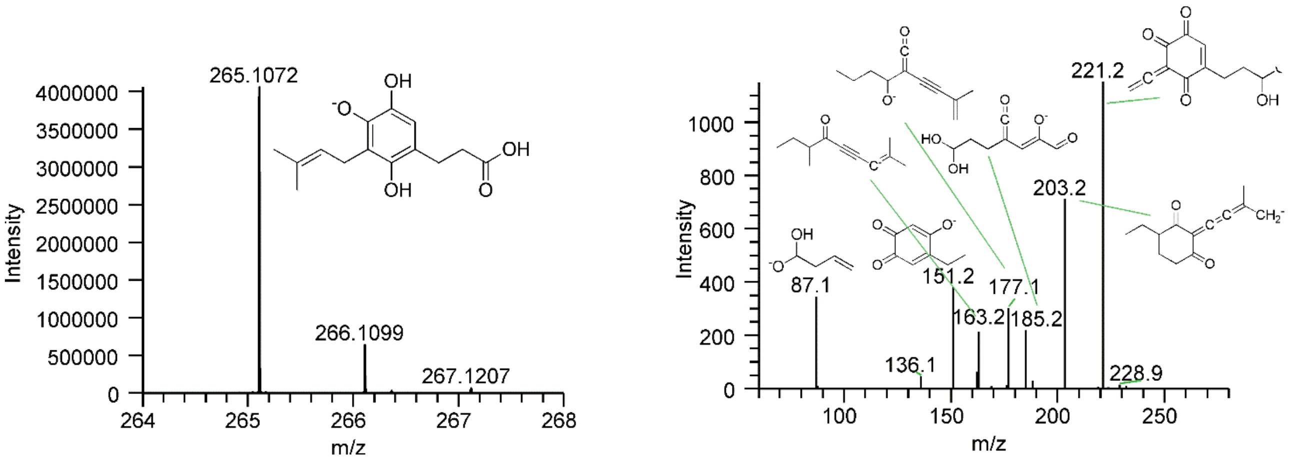

Figure S11. **3-[2,4,5-trihydroxy-3-(3-methylbut-2-en-1-yl)phenyl]propanoic acid**: $C_{14}H_{18}O_5$ *m/z* 265.1072. MS/MS: *m/z* 221, 203, 185, 177, 163, 151, 87. Found a similar structure (3-[3,4-dihydroxy-5-(3-methylbut-2-en-1-yl)phenyl]-2-hydroxypropanoic acid) through the Compound identification tool of CFM-ID. A different position of the hydroxy moiety was supposed on the base of the possibility that this structure could derive from the opening of a coumarin or the prenylation of the phenyl-

propanoic acid. The Peak Assignment tool of CFM-ID confirm an optimum match between the experimental an the in silico generated spectra.

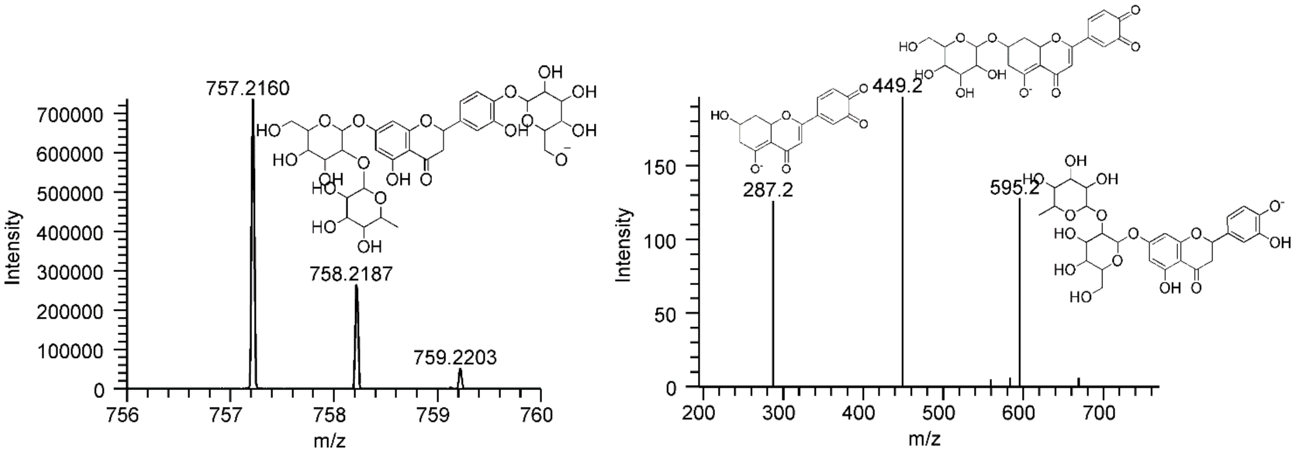

Figure S12. **Neoeriocitrin-O-glucoside/eriocitrin-O-glucoside**: $C_{33}H_{42}O_{20}$ *m/z* 757.2160. MS/MS: *m/z* 595, 449, 287. Structure hypothesized through the mass losses and confirmed by the Peak assignment tool of CFM-ID.

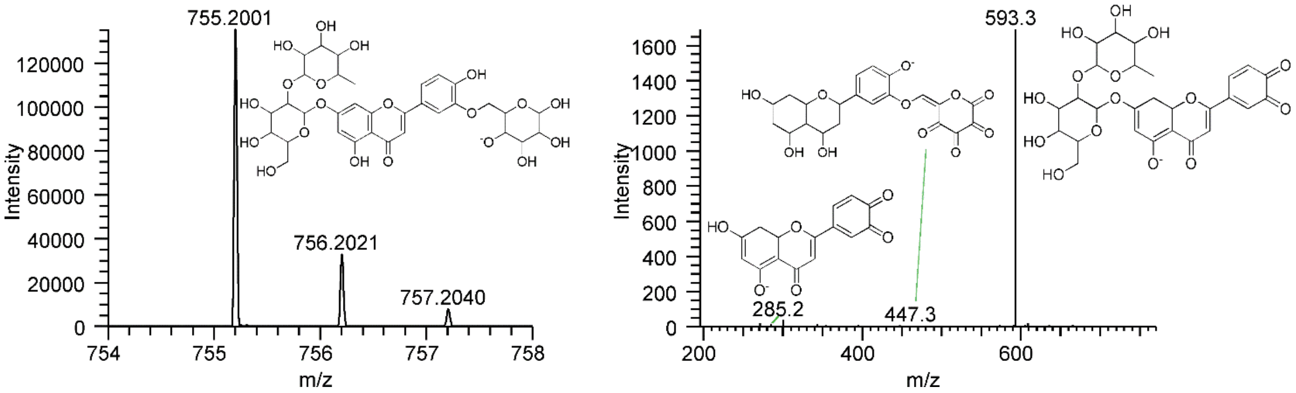

Figure S13. **Luteolin-O-neohesperidoside-O-glucoside**: $C_{33}H_{40}O_{20}$ *m/z* 755.2001. MS/MS: *m/z* 593, 447, 285. Structure hypothesized through the mass losses and confirmed by the Peak assignment tool of CFM-ID.

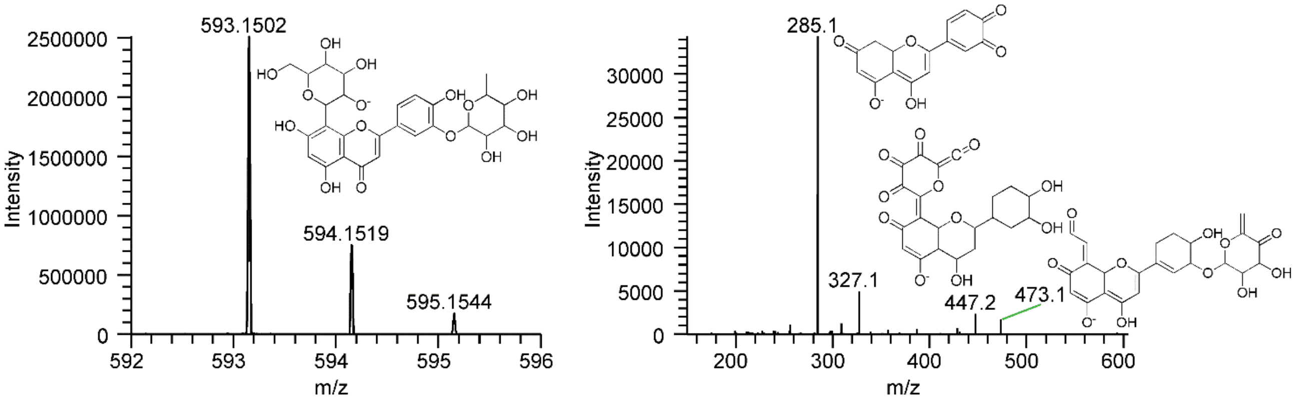

Figure S14. Luteolin-C-glucoside-O-rhamnoside: $C_{27}H_{30}O_{15}$ *m/z* 593.1502. MS/MS: *m/z* 473, 447, 285. Structure hypothesized through the mass losses and confirmed by the Peak assignment tool of CFM-ID.

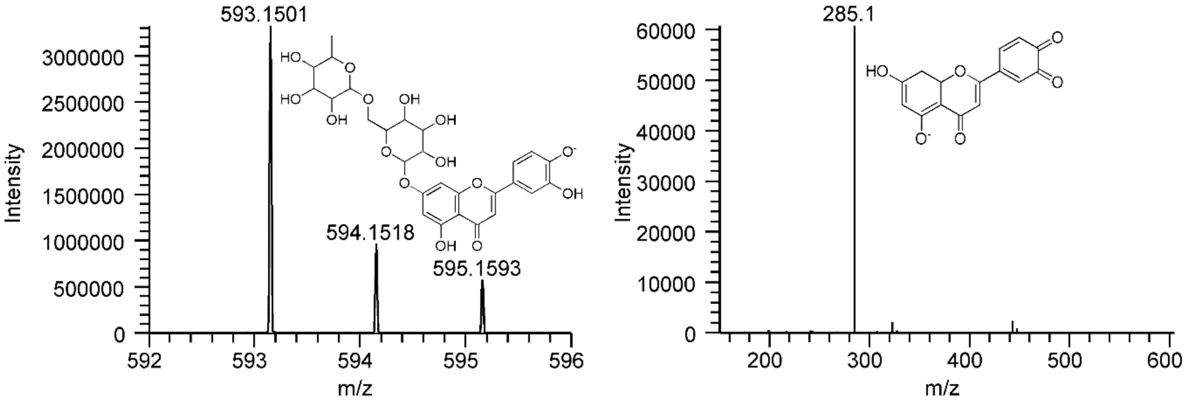

Figure S15. **Luteolin-O-rutinoside**: $C_{27}H_{30}O_{15}$ *m/z* 593.1501. MS/MS: *m/z* 285. Structure hypothesized through the mass losses and confirmed by the Peak assignment tool of CFM-ID.

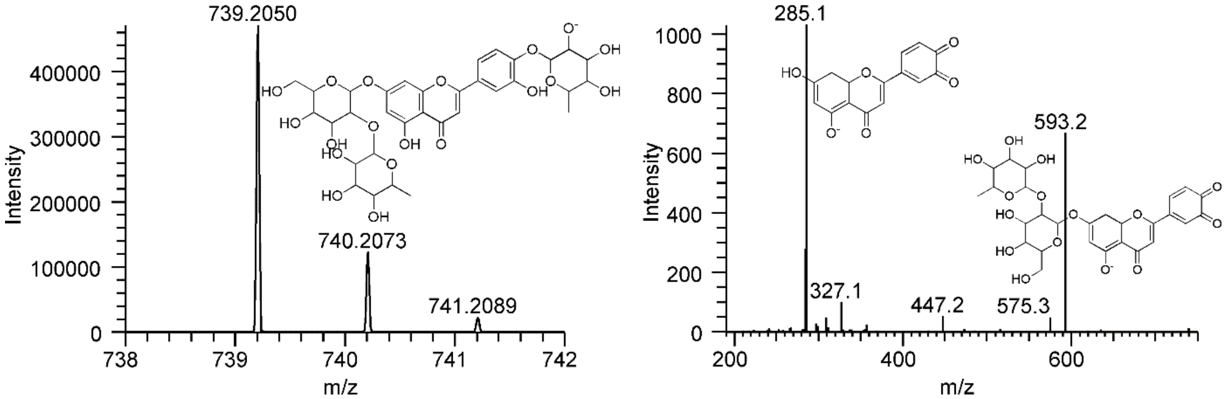

Figure S16. **Luteolin-O-neohesperidoside-O-rhamnoside**: $C_{33}H_{40}O_{19}$ *m/z* 739.2050. MS/MS: *m/z* 593, 285. Structure hypothesized through the mass losses and confirmed by the Peak assignment tool of CFM-ID.

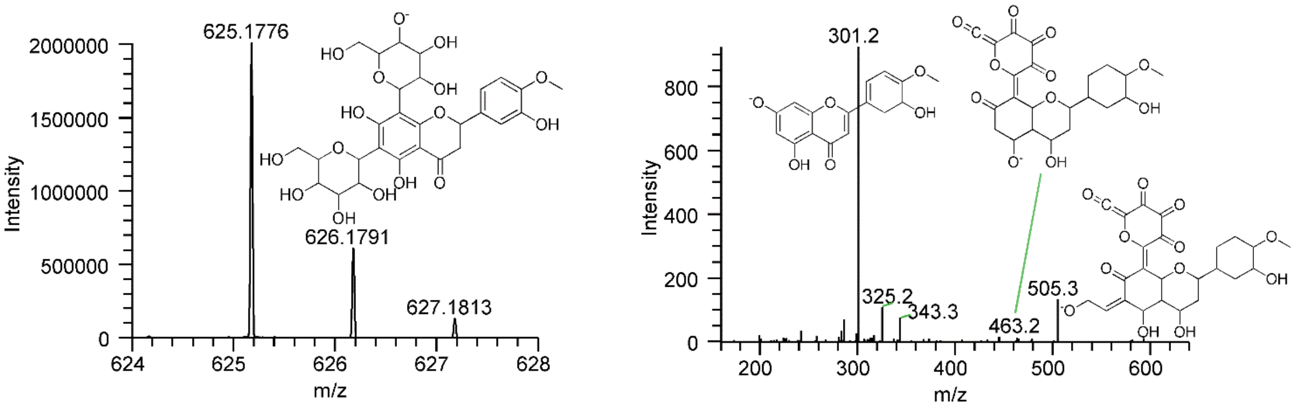

Figure S17. **Hesperetin-di-C-glucoside**: $C_{28}H_{34}O_{16}$ *m/z* 625.1776. MS/MS: *m/z* 505, 463, 343, 301. Structure hypothesized through the mass losses and confirmed by the Peak assignment tool of CFM-ID.

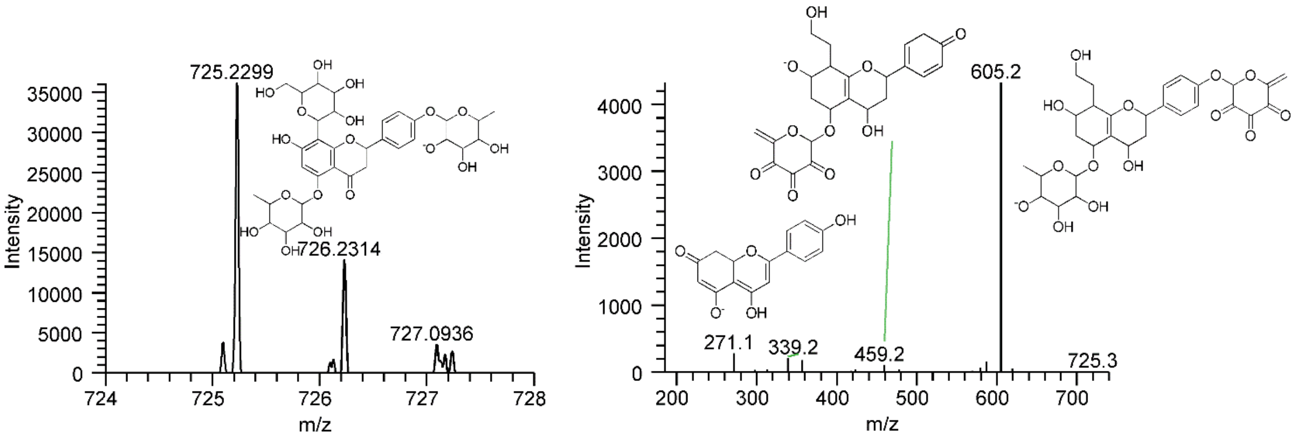

Figure S18. **Naringenin-C-glucoside-di-O-rhamnoside**: $C_{33}H_{42}O_{18}$ *m/z* 725.2299. MS/MS: *m/z* 605, 459, 271. Structure hypothesized through the mass losses and confirmed by the Peak assignment tool of CFM-ID.

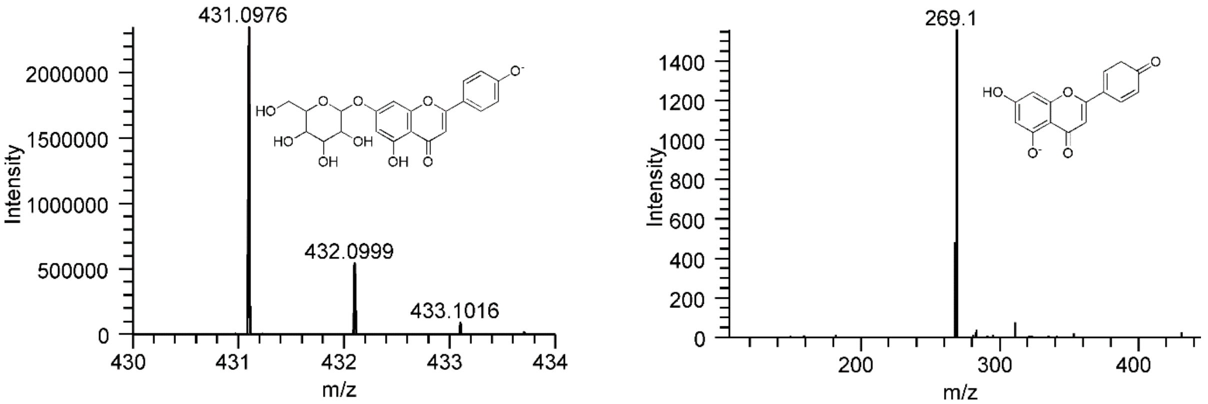

Figure S19. **Apigenin-O-glucoside:** $C_{21}H_{20}O_{10}$ *m/z* 431.0976. MS/MS: *m/z* 269. Structure hypothesized through the mass losses and confirmed by the Peak assignment tool of CFM-ID.

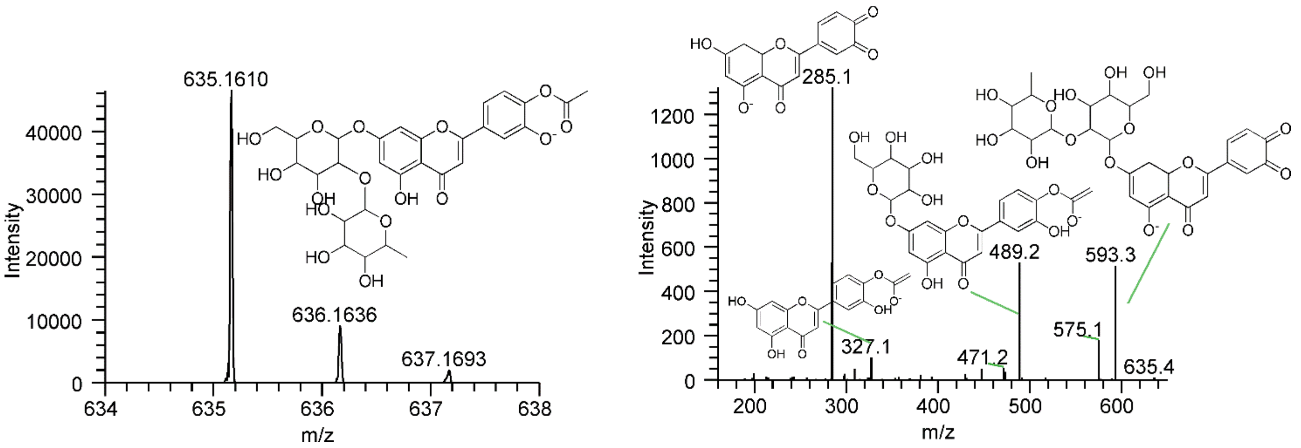

Figure S20. **Luteolin-O-acetyl-O-neohesperidoside**: $C_{29}H_{32}O_{16}$ *m/z* 635.1610. MS/MS: *m/z* 593, 489, 327, 285. Structure hypothesized through the mass losses and confirmed by the Peak assignment tool of CFM-ID.

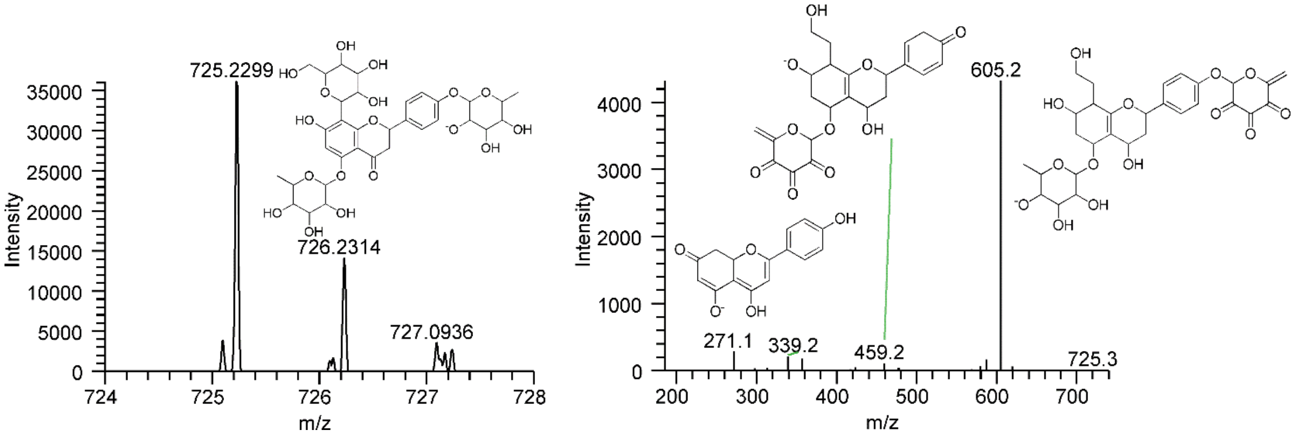

Figure S21. **Naringenin-C-glucoside-di-O-rhamnoside**: $C_{33}H_{42}O_{18}$ *m/z* 725.2299. MS/MS: *m/z* 605, 459, 271. Structure hypothesized through the mass losses and confirmed by the Peak assignment tool of CFM-ID.

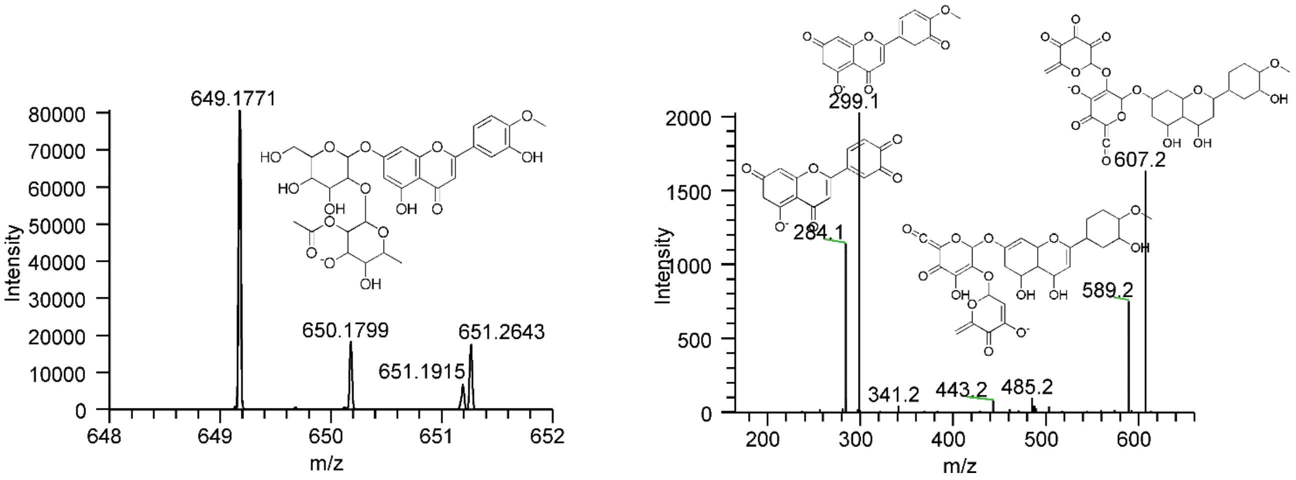

Figure S22. **Diosmetin-O-acetyl-neohesperidoside**: $C_{30}H_{34}O_{16}$ *m/z* 649.1771. MS/MS: *m/z* 607, 299, 284. Structure hypothesized through the mass losses and confirmed by the Peak assignment tool of CFM-ID.

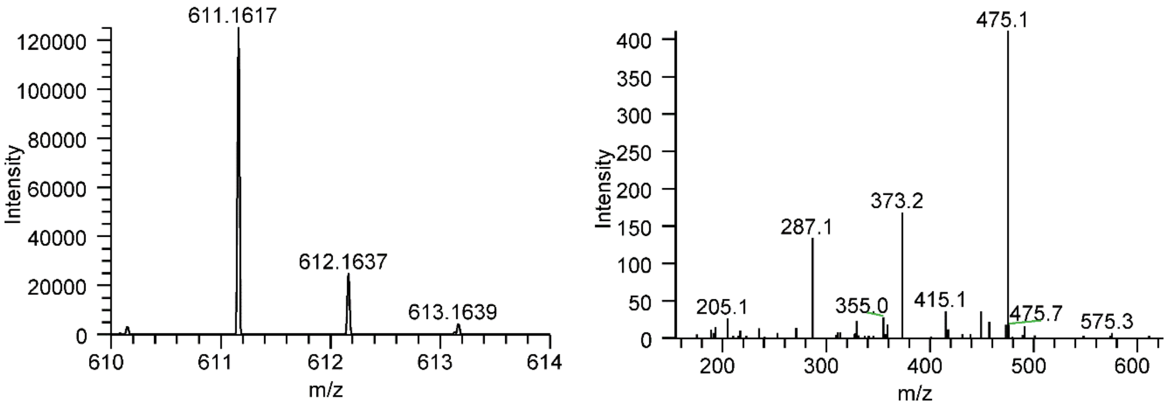

Figure S23. **Unknown 1**: $C_{27}H_{32}O_{16}$ *m/z* 611.1617. MS/MS: *m/z* 475, 373, 287.

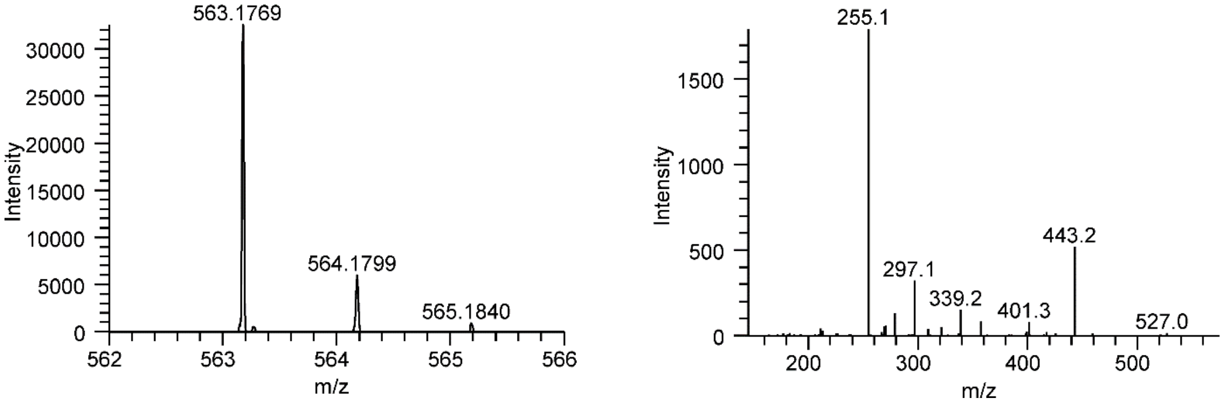

Figure S24. **255-C-glucoside-O-rhamnoside**: $C_{27}H_{32}O_{13}$ *m/z* 563.1769. MS/MS: *m/z* 443 (M-120, typical of C-glucoside), 401 (M-162, loss of the glucoside moiety; the lower intensity respect to the ion *m/z* 443 indicate a C-bond), 297 (M-120-146, loss of the rhamnoside moiety from the ion m/z 443), 255 (M-162-146, loss of the rhamnoside moiety from the ion m/z 401).

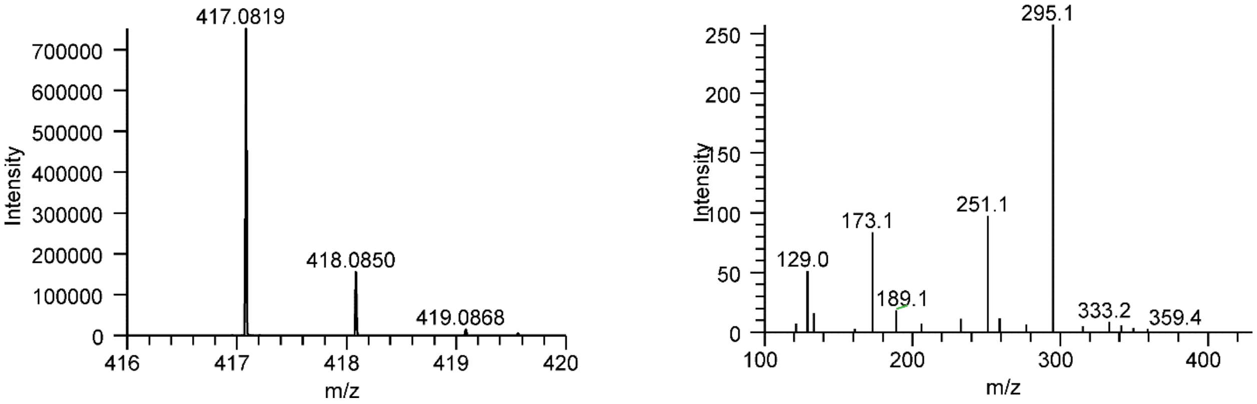

Figure S25. Unknown 2: $C_{20}H_{18}O_{10}$ *m/z* 417.0819. MS/MS: *m/z* 295, 277, 251, 189, 173, 129.

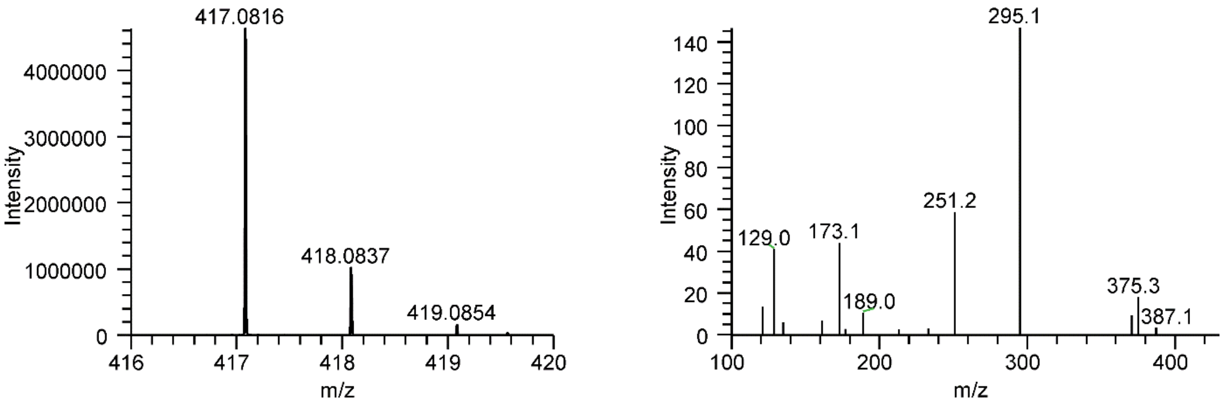

Figure S26. **Unknown 3**: $C_{20}H_{18}O_{10}$ *m/z* 417.0816. MS/MS: *m/z* 295, 277, 251, 189, 173, 129.

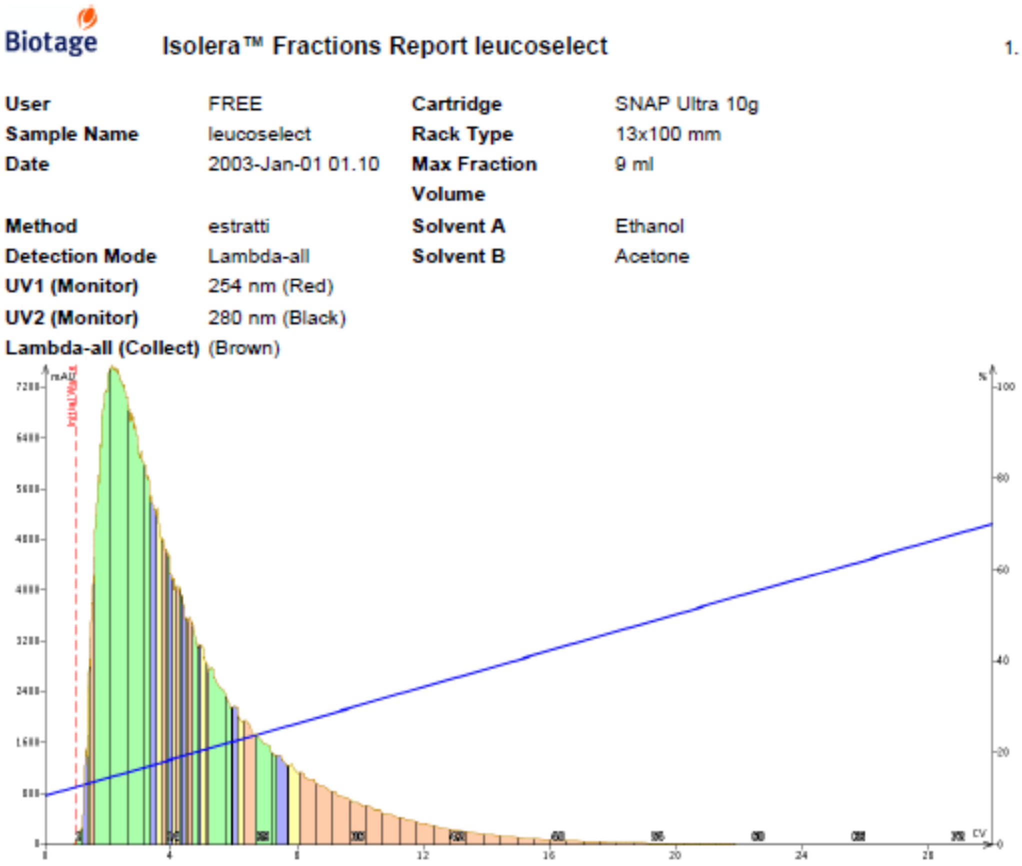

Figure S27. UV trace of the Leucoselect™ GPC. Proanthocyanidins monomers, dimers, trimers and oligomers eluted from fraction 5 up to fraction 39 over 73 fractions collected.

Biotage **Isolera™ Archive Report - 2003-Jan-01 00.23** 1.

**User**	FREE		
**Sample Name**	2003-Jan-01 00.23		
**Date**	2003-Jan-01 00.56		
**Method**			
**Project**	DPP-CA		
**Cartridge**	SNAP Ultra 10g	**Detection Mode**	Lambda-all
**Flowrate**	5 ml/min	**Baseline Correction**	On
**Solvent A**	Ethanol	**UV1 (Monitor)**	254 nm (Red)
**Solvent B**	Acetone	**UV2 (Monitor)**	280 nm (Black)
		**Lambda-all (Collect)**	(Brown)
**Rack Type**	13x100 mm	**Collect All**	On
**Max Fraction Volume**	9 ml		
**Dispense Order**	Z		

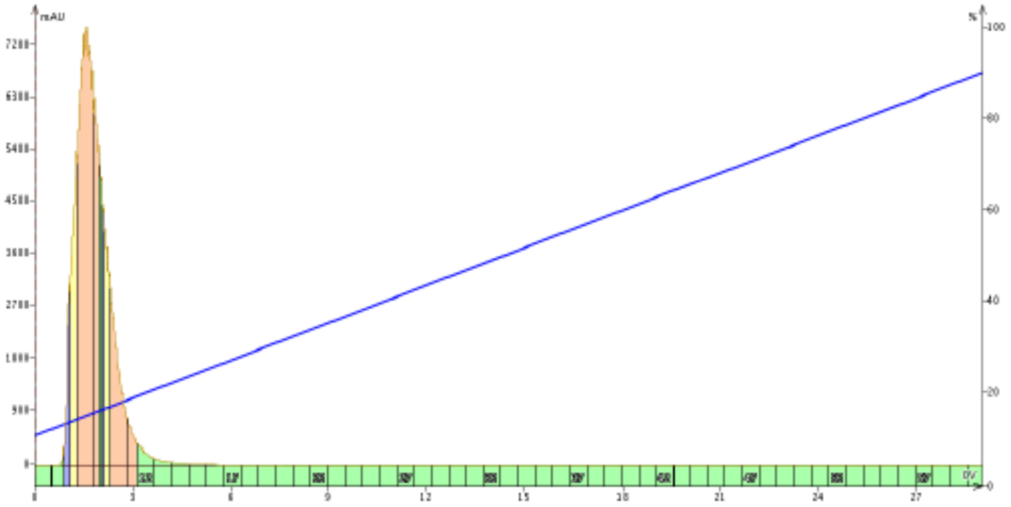

Figure S28. UV trace of the BFPF GPC. Proanthocyanidins were not detected in the fractions collected.

Biotage **Isolera™ Archive Report - 2003-Jan-01 00.39** 1.

**User**	FREE		
**Sample Name**	2003-Jan-01 00.39		
**Date**	2003-Jan-01 01.03		
**Method**			
**Project**	DPP-CA		
**Cartridge**	SNAP Ultra 10g	**Detection Mode**	Lambda-all
**Flowrate**	5 ml/min	**Baseline Correction**	On
**Solvent A**	Acetone	**UV1 (Monitor)**	254 nm (Red)
**Solvent B**	Ethanol	**UV2 (Monitor)**	280 nm (Black)
		**Lambda-all (Collect)**	(Brown)
**Rack Type**	13x100 mm	**Collect All**	On
**Max Fraction Volume**	9 ml		
**Dispense Order**	Z		

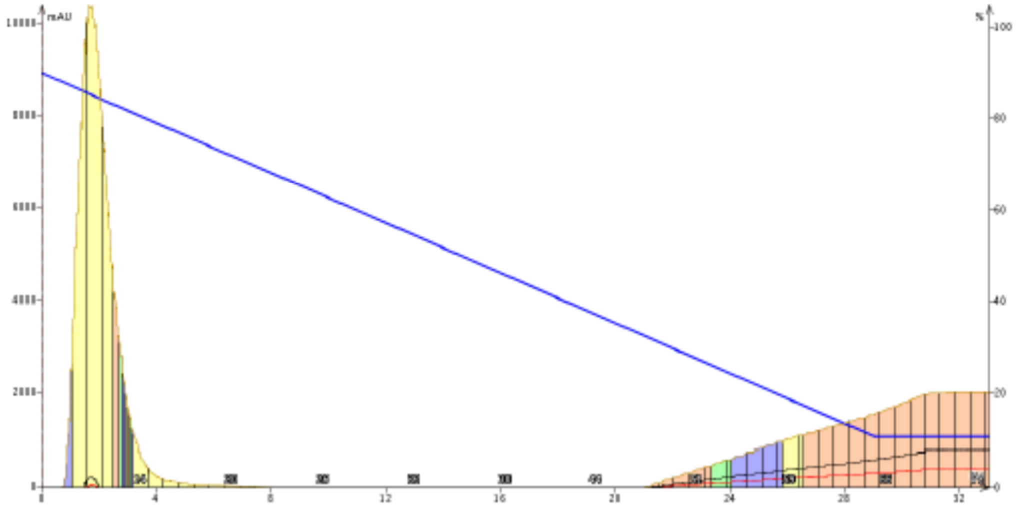

Figure S29. UV trace of the BLPF GPC. Proanthocyanidins were not detected in the fractions collected.

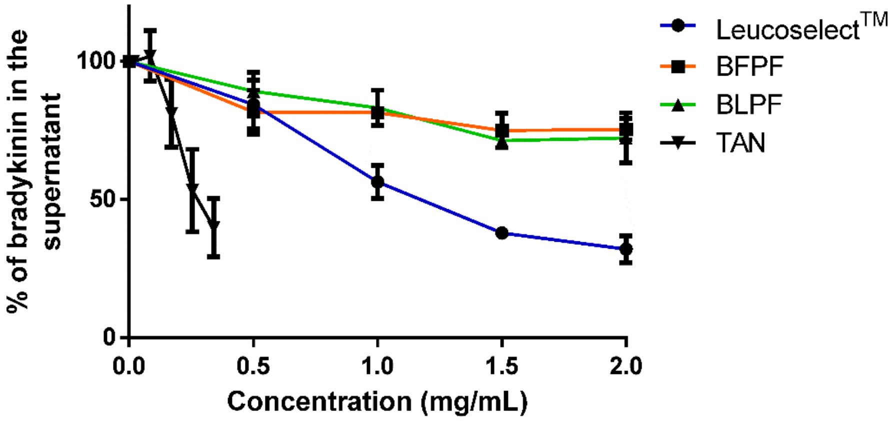

Compound/Extract	IC_{50}
Acido Tannico	114.3 μM*
BFPF	N/A
BLPF	N/A
LeucoselectTM	1.2 mg/mL

Figure S30. Evaluation of tannin effect. Bradykinin is only precipitated by tannic acid and LeucoselectTM (rich in proanthocyanidins). The IC_{50} was calculated as the concentration able to precipitate 50% of bradykinin 100 μM. *result in line with that obtained by Baron et al. [27], 112.3 μM.
